# Micro- and Nanoplastics and Functional Nutrients in Human Health: Epigenetic Mechanisms and Cellular Resilience Signaling in Brain Insulin Resistance and the Risk of Alzheimer’s Disease

**DOI:** 10.3390/ijms27010169

**Published:** 2025-12-23

**Authors:** Cinzia Lombardo, Nicolò Musso, Paolo Giuseppe Bonacci, Gabriella Lupo, Carmelina Daniela Anfuso, Eleonora Di Fatta, Raffaele Ferri, Miroslava Majzúnová, Maria Concetta Scuto, Angela Trovato Salinaro

**Affiliations:** 1 Department of Biomedical and Biotechnological Sciences, School of Medicine, University of Catania, 95123 Catania, Italy; cinzia.lombardo@unict.it (C.L.); paolo.bonacci@phd.unict.it (P.G.B.); gabriella.lupo@unict.it (G.L.); daniela.anfuso@unict.it (C.D.A.); 2 Department of Medicine and Surgery, Kore University of Enna, 94100 Enna, Italy; nicolo.musso@unikore.it; 3 Advanced and Innovative Diagnostic Academy (A.I.D.A.) S.r.l., Spin-Off of BRIT Research Center, University of Catania, 95125 Catania, Italy; 4 OASI Research Institute-IRCCS, 94018 Troina, Italy; edifatta@oasi.en.it (E.D.F.); rferri@oasi.en.it (R.F.); 5 Department of Animal Physiology and Ethology, Faculty of Natural Sciences, Comenius University, Ilkovicova 6, 84215 Bratislava, Slovakia; miroslava.majzunova@uniba.sk; 6 Institute of Normal and Pathological Physiology, Centre of Experimental Medicine, Slovak Academy of Sciences, Sienkiewiczova, 84104 Bratislava, Slovakia

**Keywords:** microplastics, nanoplastics, functional nutrients, neurons, glial cells, brain, *NFE2L2* gene, epigenetic mechanisms, ferroptosis, oxidative stress, inflammation, antioxidant enzymes, diabetes, brain insulin resistance, Alzheimer’s disease

## Abstract

The therapeutic potential of functional nutrients has garnered considerable attention for enhancing resilience signaling and counteracting the damage to human health caused by microplastic pollutants. The intricate interactions between microplastics (MPs) and nanoplastics (NPs) and functional nutrients, including polyphenols, flavonoids, phenylpropanoids, phenolic acids, diterpenoids, and triterpenoids, have been shown to improve blood–brain barrier (BBB) homeostasis and brain function by inhibiting oxidative stress, ferroptosis, and inflammation linked to the pathogenesis of metabolic and brain disorders. Interestingly, nutrients exhibit biphasic dose–response effects by activating the nuclear factor erythroid 2-related factor 2 (Nrf2) pathway and stress-resilience proteins at minimum doses, thereby preventing or blocking MP and NP-induced damage. Notably, chronic exposure to environmental pollutants causes aberrant regulation of *NFE2L2* gene and related antioxidant signaling, which can exacerbate selective susceptibility to brain insulin resistance under inflammatory conditions. This, in turn, impairs glucose metabolism and facilitates β-amyloid (Aβ) plaque synthesis leading to the onset and progression of Alzheimer’s disease (AD), also known as “Type 3 diabetes”. This pathological process triggered by oxidative stress, inflammation, and ferroptosis creates a vicious cycle that ultimately contributes to neuronal damage and loss. The review aims to investigate the therapeutic potential of functional nutrients targeting the Nrf2 pathway and stress resilience proteins to regulate epigenetic alterations, and to explore the underlying molecular mechanisms using innovative in vitro platforms for the development of promising preventive strategies and personalized nutritional interventions to attenuate oxidative stress, ferroptosis, and inflammation, with the goal of ultimately improving clinical outcomes.

## 1. Introduction

The increasing presence of microplastics and nanoplastics (MNPs) in the environment has raised significant concerns about their potential impact on human health. Human exposure to MNPs occurs through ingestion of contaminated food and beverages, as well as inhalation of particles released from textiles and plastics [1]. MPs are defined as plastic particles < 5 mm in diameter [2]. After additional erosion, MPs become NPs, particles with at least one dimension < 1 mm [3]. According to various studies, polyethylene terephthalate (PET), polypropylene (PP), polyethylene (PE), polyvinyl chloride (PVC), polycarbonate (PC), polyamide (PA), and polystyrene (PS) particles are the most frequently present in foods [1]. These particles can cross significant physiological barriers. Indeed, MPs have been detected in various human tissues, such as the lung, liver, intestine, kidney, and brain, suggesting that they can enter and distribute within the body, resulting in toxic effects, including neurotoxicity, metabolic toxicity, and carcinogenesis [4]. In vitro evidence has identified a variety of toxic effects caused by MNPs exposure on mammalian cells, including cytotoxicity, oxidative stress, endoplasmic reticulum stress, apoptosis, mitochondrial dysfunction, inflammatory response, and genotoxicity [5]. Accumulation and translocation of MNPs have also been confirmed in vital organs like the liver, spleen, and lymphatic system of animal models [6]. Alzheimer’s disease (AD) is a progressive neurodegenerative disorder and one of the leading causes of dementia in the world. A study based on the Global Burden of Disease (GBD) 2019 analysis projects that the number of people living with dementia globally will rise from an estimated 57.4 million in 2019 to 152.8 million in 2050 [7]. Recently, there has been a growing interest in the scientific community regarding the potential connection between AD and type 2 diabetes mellitus (T2DM), leading to the proposal of the conceptual term “type 3 diabetes mellitus” (T3DM) [8,9]. This term is intended to underscore the potential pathophysiological role of insulin resistance in the brain and its association with Aβ and tau protein in the peripheral nervous system and related organs, linking them with the onset and progression of AD [5]. However, it is important to note that T3DM is not an officially recognized medical or diagnostic category. Insulin resistance is a central hallmark that connects T2DM to the resultant formation of advanced glycation end products (AGEs), which in turn leads to complications such as AD pathogenesis. It involves altered insulin mechanisms and cellular pathways including the mitogen-activated protein kinases (MAPK), extracellular signal-regulated kinases 1 and 2 (ERK1/2), c-Jun N-terminal kinase (JNK1-3), Nrf2, p38, glycogen synthase kinase 3 β (GSK3β), mammalian target of rapamycin (mTOR), forkhead box O (FOXO), phosphoinositide 3-kinase (PI3K) and protein kinase B (Akt) [9]. Compelling studies report that altered metabolic function and glucose metabolism may lead to abnormalities that cause progressive brain insulin resistance with consequent impairment of central insulin signaling processes and the development of T3DM [10]. Brain insulin resistance triggers a cascade of damage in AD by disrupting insulin signaling, leading to mitochondrial dysfunction, increased oxidative stress and neuroinflammation, which collectively promote tau hyperphosphorylation and Aβ accumulation, ultimately causing synaptic failure and neuronal death, and essentially making AD a “brain diabetes” [10]. Increasing attention is being paid to the adverse effects of MP ingestion by organisms, yet the potential ramifications on mammalian blood glucose levels, including T2DM and the risk of AD initiation and progression, remain unexplored. Emerging studies have indicated that MPs can cause abnormal glucose and lipid metabolism [11]. Consistent with this, Shi et al. indicated that exposure to PS-MPs, especially with a diameter of 1 μm or less, induced an increase in insulin resistance [12] and aggravated glucose tolerance [13] in mice via gut-liver axis metabolic disturbances. Furthermore, exposure to the polystyrene nanoplastics (PS-NPs-NH_2_) group can inhibit the phosphorylation of AKT and FoxO1, which results in altered blood glucose levels and T2DM-like lesions [14]. Similarly, polystyrene microplastics (PS-MPs) (0.5 µm) significantly increased oxidative stress and elevated levels of NOD-like receptor protein 3 (NLRP3)/caspase-1 and TGF-β1/Smads signaling pathways, exacerbating renal tissue damage and fibrosis in db/db mice [15]. Oxidative stress triggers excessive production of free radicals and depletion of cellular detoxifying phase II systems, causing damage to proteins, lipids, and DNA, as well as induction of genetic mutations in DNA/RNA [16]. Reactive oxygen species (ROS) alter brain glucose metabolism and promote the production of pro-inflammatory cytokines, leading to the development of metabolic syndrome [17] and AD in humans [14,15]. Nrf2 is a redox-sensitive transcription factor encoded by the *NFE2L2* gene, which is also the master regulator of phase II antioxidant enzymes that protect against oxidative stress and inflammation [18]. Under physiological conditions, Nrf2 is sequestered by Kelch-like erythroid cell-derived protein with CNC homology (ECH)-associated protein 1 (Keap1) in a complex in the cytoplasm, where its level is regulated by ubiquitination and proteasomal degradation [17]. Under stress conditions, the interaction between the Nrf2 and Keap1 complexes is disrupted, and Nrf2 is accumulates in the cytoplasm and then translocates into the nucleus, where it binds to phase II of the antioxidant response element (ARE) and initiates the transcription of cellular resilience proteins and enzymes, particularly heat shock protein 70 (Hsp70), heme oxygenase-1 (HO-1), sirtuin-1 (Sirt1), the thioredoxin (Trx)/thioredoxin reductase system, NADPH quinone oxidoreductase 1 (NQO1), γ-glutamylcysteine synthetase (γ-GCs), superoxide dismutase (SOD), catalase (CAT), glutathione (GSH), glutathione peroxidase (GPx), glutathione reductase (GSR), and forkhead box O3 (FOXO3) to regulate redox balance and protect against the onset and progression of metabolic and neurodegenerative disorders [19,20,21]. Importantly, cellular stress resilience response activated by functional nutrients is emerging as a novel personalized nutritional strategy to prevent and treat oxidative stress and inflammatory states occurring in both metabolic and neurodegenerative disorders [18]. Functional nutrition is a holistic approach to food and nutrition that focuses on the interconnection of body systems and how food acts as information, not just fuel, to impact health. It emphasizes personalized dietary and lifestyle recommendations based on an individual’s unique biochemical makeup, considering factors like genetics, environment, and lifestyle choices. Unlike traditional nutrition, which often focuses on symptom management, functional nutrition aims to identify and address the root causes of health issues. Functional nutrients are chemical or biological components present in foods that, when introduced in adequate doses, exert beneficial physiological effects on human health. These nutrients, including polyphenols, vitamins, flavonoids, tannins, alkaloids, phenolic acids, and triterpenoids, are plant secondary metabolites widely distributed in various parts of plants that modulate and upregulate the Nrf2 signaling pathway and stress resilience proteins to preserve cellular redox homeostasis during MNP-induced damage and mitigate the risk of developing pathological processes like T2DM and AD [22]. The term “nutraceutical” was coined by Dr. DeFelice and refers to a food or part of a food in concentrated form, such as a dietary supplement, that has a medical or health benefit, including the prevention and treatment of disease, but is not essential to the diet [23,24,25]. This suggests that the terms “functional nutrients” and “nutraceutical” are often used interchangeably and that the fundamental characteristic of a nutraceutical is that it is a food without food additives [26]. The review aims to explore the current knowledge about the protective effects of functional nutrients to prevent or mitigate the oxidative stress, ferroptosis, and epigenetic mechanisms induced by MNPs exposure by targeting the Nrf2 pathway and cellular resilience proteins in order to enhance metabolic health, cognitive function, and overall well-being. Moreover, this review summarizes advanced in vitro platforms for detecting cellular MNPs, with the ultimate goal of discovering promising personalized nutritional therapeutic interventions for the prevention and management of Alzheimer’s disease and its complications. The innovative approach focuses on identifying specific MNPs associated with the onset and progression of these pathological conditions and tailoring nutritional recommendations to prevent or relieve clinical symptoms accordingly.

## 2. Brain Glucose Metabolism in AD or “Type 3 Diabetes”

**Figure 1:**
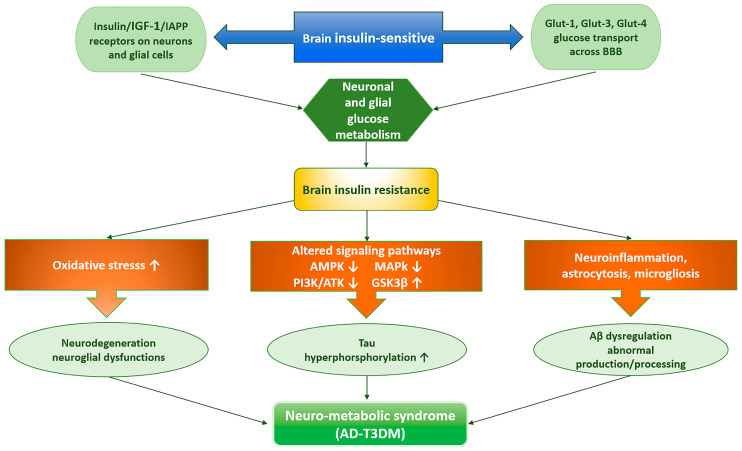
Molecular signaling cascades in neuronal insulin resistance and AD pathogenesis. The arrow ↑ is an upregulation; the arrow↓ is a downregulation.

Previously, the brain was thought to be an insulin-insensitive tissue. Currently, several lines of evidence demonstrate the presence of insulin in the brain. The concept of the brain as an insulin-sensitive organ is supported by the presence of insulin-responsive glucose transporter 4 (GLUT-4), insulin-like growth factor 1 (IGF-1), islet amyloid polypeptide (IAPP), and other receptors on the surface of neurons and glial cells. These receptors stimulate glucose uptake and metabolism in the brain [27]. The desensitization of the neuronal insulin receptor due to brain insulin resistance, similar to the process in T2DM, may play a key role in causing T3DM and its future complications, particularly abnormal Aβ expression and protein processing [28]. In the early stages of AD, brain glucose utilization is reduced, while hyperinsulinemia, a hallmark of insulin resistance, and blood glucose levels are increased. In physiological conditions, insulin can bind to insulin substrates through the BBB, thus mediating insulin-signaling pathways to regulate energy metabolism and protect neurons. At the molecular level, brain cells sense insulin substrate through insulin receptors. Thus, impaired insulin signaling and receptor function cause oxidative stress and reduce the astrocytic energy substrates and the antioxidant supply of neurons, while glucose excess (associated with hyperleptinemia) may worsen the reduced astrocytic energy supply and the ongoing neuroinflammation through the inhibition of AMP-activated protein kinase (AMPK), MAPK- and PI3K/Akt-signaling pathways, and GSK-3β pathway, ultimately leading to increased tau deposition and hyperphosphorylation (Figure 1) [27,29].

Indeed, insulin resistance induces neuroglial alterations (astrocytosis and microgliosis) that appear to contribute to obesity, T2DM, and AD neuropathology in APP + PS1 mice [27]. In the brain, glucose bioavailability is limited by the traversal of the BBB, a process mediated by glucose transporters GLUT1-6 and GLUT-8 and sodium-dependent transporters (SGLT1) to reach neurons and glial cells [30]. The GLUT1 transporter is expressed in capillary endothelial cells and transfers glucose across the BBB into astrocytes [31]. Conversely, GLUT3 is expressed predominantly in neurons and exhibits high affinity for glucose [32], and GLUT4 is expressed in the BBB of the ventromedial hypothalamus, hippocampus [33] and the temporal cortex; therefore, it actively participates in memory and cognition processes [34]. Both GLUT1 and GLUT3 are insulin-independent for membrane translocation [35]. However, GLUT3 and GLUT4 transporters’ expression decreases with aging, which may be due to increased levels of inflammatory markers (Figure 1) [36]. Overall, the data strongly suggest that impaired insulin signaling and brain glucose metabolism may represent the potential link between metabolic syndrome and the onset and progression of AD pathogenesis, also known as “neurometabolic syndrome.”

### 2.1. Molecular Mechanisms Linking T2DM to AD

#### 2.1.1. Cerebrovascular Damage in T2DM and AD

**Figure 2:**
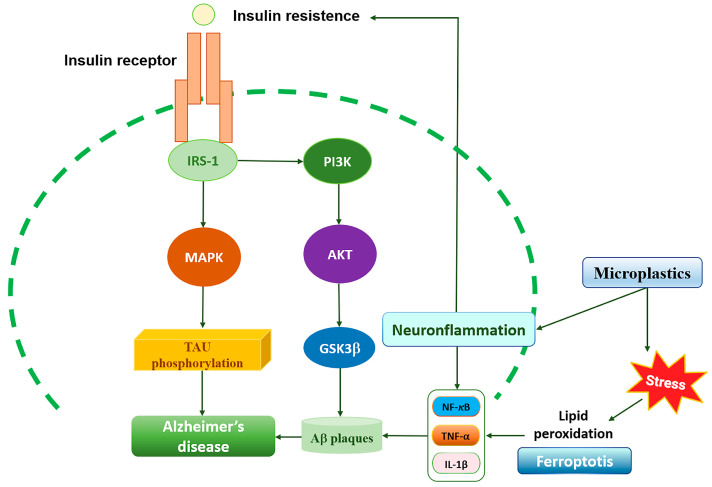
Molecular pathways at the neuronal level involved in the development of AD that inhibit insulin signaling by blocking the insulin receptor substrate and the PI3K/AKT cascade.

Chronic hyperglycemia and insulin resistance in T2DM damage blood vessels, causing cognitive impairment through a process that involves oxidative stress, inflammation, and endothelial dysfunction. This damage leads to various cerebrovascular complications, such as atherosclerosis, which can block blood flow to the brain, increasing the risk of ischemic stroke, memory loss, and cognitive decline. Interestingly, growing evidence suggests that cerebrovascular disease contributes to cognitive impairment in diabetic patients [37]. This occurs because dysregulation of cerebrovascular function in diabetes can severely impact brain perfusion and function and the removal of metabolites from the brain. Specifically, high blood glucose alters brain vessel integrity and elasticity. Furthermore, T2DM may impair the vascular-mediated Aβ clearance system and thus contribute to Aβ deposition in the brain. An imbalance between Aβ production and clearance initiates AD by promoting Aβ accumulation in the brain (Figure 2) [38]. The early-onset, or familial, form of AD is often caused by genetic mutations (e.g., APP, presenilin 1 and 2) leading to Aβ overproduction, while the more common sporadic (late-onset) form is believed to be caused by impaired Aβ clearance [38]. Overall, cerebrovascular disease could be a common mechanism linking T2DM and AD.

#### 2.1.2. IGF-1 and GSK3β Pathways Linking Brain Insulin Resistance to Type 3 Diabetes

Growing evidence supports that impaired insulin signaling in the brain may be responsible for early and progressive cognitive defects in patients with T2DM and AD [9]. In physiological conditions, insulin/IGF signaling promotes the trafficking of AβAPP-Aβ [39,40] and also enhances clearance of Aβ by modulating Aβ transporters and carriers at the BBB [41]. In addition, insulin and IGF-1 inhibit the activation of GSK3β by phosphorylation on serine 9 via the PI3K/Akt pathway, thus limiting its ability to phosphorylate tau and promoting the binding of tau to microtubules, maintaining neuronal stability [42]. However, when insulin/IGF signaling is dysregulated, it increases AβAPP deposition, tau hyperphosphorylation, ROS, loss of synaptic plasticity, neuroinflammation, and ultimately decreases cerebral blood flow [43]. Accordingly, AβPP-Aβ oligomers inhibit neuronal insulin-stimulated signals and block PI3K activation of Akt, which leads to impaired survival signaling, increased activation of GSK-3β (Figure 2), resulting in the hyperphosphorylation of tau leading to the formation of neurofibrillary tangles (NFT). This supports the notion that AβPP-Aβ promotes oxidative stress, antioxidant depletion, mitochondrial and synaptic dysfunction, ultimately contributing to brain insulin resistance and the onset of T3DM [44]. Several studies of postmortem brains of AD patients have indicated that insulin/IGF-1 resistance, along with aberrant activation of the PI3K/Akt pathway, leads to the overactivation of GSK-3β and reduced insulin/IGF-1 levels (which act as neurotrophic factors) [10,45,46,47]. These abnormalities are more severe in brain regions involved in cognitive performance, particularly in the hippocampus, due to its crucial role in cognitive functions like memory and learning [48], but also in the frontal, parietotemporal, and cingulate cortices [49], indicating that insulin resistance affects the same regions as those affected by AD. This suggests a link between central insulin resistance and T3DM. Of note, GSK3 negatively regulates Wnt signaling, which is an important pathway involved in synaptic plasticity mechanisms. During brain insulin resistance, GSK3 is hyperactivated, which impairs the Wnt signaling pathway by degrading β-catenin. This leads to alterations in synaptic plasticity and memory function in the prefrontal cortex, contributing to cognitive decline observed in AD patients [50]. Similarly, Aβ peptides can also interfere with insulin and Wnt signaling pathways, which leads to GSK3 dysregulation, thus establishing a link between senile plaques and the formation of NFT in AD [51]. Functional nutrients have potential therapeutic benefits in mitigating insulin resistance and the risk of cognitive dysfunction. Consistent with this, a comparative study evaluated the capacity of mulberry anthocyanin extract on insulin resistance in vitro and in vivo [52]. Specifically, mulberry anthocyanin extract inhibited the effect of high glucose in hepatocellular carcinoma cells by targeting the activation of the PI3K/AKT pathway (Table 1). Likewise, supplementation of anthocyanins (50 and 125 mg/kg per day) lowered hepatic glycogen content, resulting in changes to the phosphorylation of GSK3β and FOXO1 in the liver of db/db mice [52]. Overall, the data indicate that brain insulin resistance and altered IGF-1 signaling disrupt the PI3K/AKT pathway, leading to the activation of GSK3β, which in turn promotes tau hyperphosphorylation and AD pathology (Figure 2). This suggests that nutritional medicine targeting PI3K/AKT and GSK3β signaling could represent a potential preventive and regenerative therapy to minimize the impact of extrinsic mediators on aging transitions (e.g., inflammatory states, oxidative stress, obesity, systemic insulin resistance) while also implementing neuroprotective measures in both T2DM and AD.

#### 2.1.3. Brain Insulin Resistance and the Formation of Aβ and IAPP Peptides

Emerging evidence suggests that AD and T2DM share a common pathological mechanism called amyloidosis, where protein plaques build up and cause cell death [53]. In AD, the Aβ peptide, primarily Aβ1-40 and Aβ1-42 subtypes, aggregates into plaques in the brain. These plaques disrupt normal brain function, causing neuroinflammation, synaptic dysfunction, and eventually neuronal loss, leading to cognitive decline. Similarly, in T2DM, pancreatic β cells secrete islet amyloid polypeptide (IAPP) or amylin, which can misfold and aggregate into neurotoxic amyloid plaques in the islet of Langerhans [53]. Biologically, unmodified IAPP regulates brain functions such as appetite and cognition. Also, it crosses the BBB and enhances the clearance of the Aβ1-42 subtype to potentially prevent the development and progression of AD [54]. However, oxidative stress modifies IAPP, leading to loss of its normal neuroprotective function, promoting its aggregation into toxic amyloid deposits involved in AD neuropathology (Figure 2). This suggests that Aβ formation is a hallmark in both AD and T2DM due to the deposition and aggregation of circulating IAPP in both pancreatic β islets and the brain [53]. Abnormal immune responses to the aggregated IAPP trigger the release of inflammatory cytokines and the expression of nuclear factor-κB (NF-κB) inflammatory signaling (Figure 2). This inflammatory cascade amplifies IAPP-induced toxicity in both T2DM [55] and AD [56]. Therefore, Aβ neurotoxicity is potentially mediated by the action of IAPP. More specifically, IAPP from the pancreas can cross the BBB and may interact with Aβ, potentially accelerating AD progression [54]. Consistent with this, a study performed on postmortem brain tissues of AD patients, in conjunction with in vitro and experimental mouse models, reported that IAPP promotes tau protein deposition, accompanied by more severe synapse loss and cognitive deficits [57]. Overall, these data support a link between tau and IAPP amyloid, which seems to act coordinately to impair β-pancreatic cell function and glucose homeostasis, and suggest that the combined pathological actions of these proteins may be a potential mechanism connecting T2DM and AD.

**Table 1:** Molecular targets and neurotoxic effects related to neuronal insulin receptor desensitization in brain insulin resistance.

Molecular Target	Type of Alteration	Neurotoxic Effects	Ref.
Neuronal insulin receptor	Desensitization and brain insulin resistance	Reduced insulin signaling and increased oxidative stress, neuroglial alterations, tau deposition, and hyperphosphorylation	[27,28,29]
AMPK	Inhibition	Worsening of astrocytic energy deficiency and neuroinflammation	[27,29]
MAPK	Inhibition	Altered cellular stress response and reduced neuronal protection	[27,29]
GSK-3β	Activation	Increased tau protein deposition and hyperphosphorylation	[27,29]
Astrocytes	Reduced energy and antioxidant supply	Increased neuronal oxidative stress and neuroinflammation	[27,29]
Microglia	Activation (microgliosis)	Chronic neuroinflammation and progression of AD neuropathology	[27]
Aβ	Increased expression and altered processing	Formation of amyloid plaques typical of AD	[28]
GLUT1	Impaired glucose transporter	Reduced glucose transport across the BBB and decreased energy availability for astrocytes	[30,31,35]
SGLT1	Reduced transport efficiency	Lower glucose bioavailability for neurons and glial cells	[30]
BBB	Limited insulin and glucose transport	Altered cerebral energy metabolism and increased neuronal vulnerability	[30,31]
GLUT3	Reduced expression with aging	Reduced glucose availability to neurons, neuronal energy deficit	[32,35,36]
GLUT4	Reduced expression with aging and inflammation	Reduced glucose transport, impairment of memory, and cognitive functions	[33,34,35]
Aβ	Impaired Aβ clearance	Dysregulated cerebrovascular function, altered brain vessel integrity, with accumulation of Aβ in the brain	[37,38]
IGF-1	Altered receptor function	Increased AβAPP deposition and tau hyperphosphorylation, and decreased cerebral blood flow	[43]
PI3K/AKT	Inhibition	Reduced neuronal survival, increased neuroinflammation, and activation of GSK-3β	[44,45,46,47]
Wnt	Inhibition	Altered synaptic plasticity and memory function in the prefrontal cortex, contributing to cognitive decline	[50]
IAPP	Aggregation into toxic amyloid deposits	Altered cerebral glucose metabolism, increased pro-inflammatory cytokines and tau protein deposition accelerate AD progression	[53,54,55,56,57]

## 3. Functional Nutrition Targeting Cellular Resilience Signaling Improves Brain Insulin Resistance and the Risk of Alzheimer’s Disease

**Figure 3:**
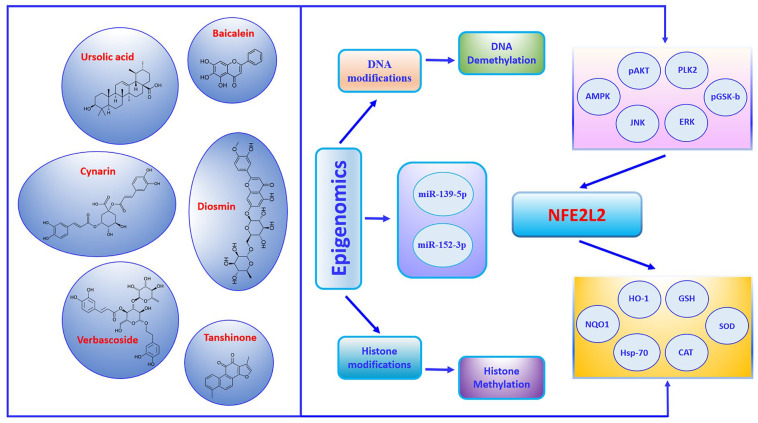
Schematic representation of epigenetic mechanisms regulated by functional nutrients. Activation of the *NFE2L2* gene and stress resilience proteins prevents or reverses epigenetic modifications.

Nutritional medicine plays an essential role in regulating blood sugar and improving cognitive function in the treatment of both T2DM and AD. Specifically, certain nutrients/polyphenols (e.g., ursolic acid, verbascoside, diosmin, cynarin, baicalein, and tanshinone) can regulate insulin resistance and neuroinflammation, and normalize the function of neuroglial cells (preventing and/or reversing neurotoxic responses) to enhance brain homeostasis in particular synaptic plasticity, dendritic arborization, neurotransmitter expression, neuronal survival, signal transduction, learning and memory function by modulating key signaling pathways. These pathways include the upregulation of Nrf2, AMPK, and PI3K/Akt and the downregulation of GSK3β and MAPK, all of which have been implicated in AD when altered (Figure 3) [58,59]. Given their overlapping pathways with insulin signaling, functional nutrition may offer novel avenues for preventing and managing AD [60]. By understanding how ursolic acid, verbascoside, diosmin, cynarin, baicalein, and tanshinone modulate Type 3 diαβetes (AD), personalized dietary interventions (e.g., an adequate dose of specific nutrients or dietary supplements) can be implemented to improve metabolic health, cognitive function, and overall well-being. Interestingly, food nutrients and nutraceutical ingredients have been proven to protect against the progression of disease by inhibiting 5α-reductase activity, IL-6 secretion, and the lipid peroxidation process [61]. Notably, polyphenolic compounds exhibit an anti-AD potential from the perspective of T3DM. Recent findings indicate that a tannin-enriched fraction of TeMac™ (400 mg/kg) administered by daily gavage for 42 days prevents the formation of senile plaques. This is due to its ability to inhibit β-site amyloid precursor protein cleaving enzyme 1 (BACE-1) and the activities of β-secretase and monoamine oxidase A, as well as Aβ fibrillation. Conversely, it activates the antioxidant enzyme CAT, ultimately reversing aluminum chloride-induced insulin resistance and AD-like pathology in diabetic rats. Therefore, the tannin-enriched fraction of TeMac™ may be a potential drug candidate for the treatment of diabetes-associated cognitive impairment [22]. Furthermore, wood extracts rich in hydrolyzable tannins upregulate SGLT1 and GLUT2 expression at 4 µg/mL, glucose uptake at 1 µg/mL, and GLUT4 expression at 12 µg/mL, respectively, in the 3D intestinal cell model [62]. Moreover, natural exosome-like nanoparticles from mung bean sprout juice reduce oxidative stress levels in liver tissue by upregulating GLUT4 and the Nrf2 pathway and related antioxidant enzymes, such as HO-1 and SOD, and downregulating GSK-3β via dose-dependent and time-dependent activation of the PI3K/Akt signaling pathway in T2DM animal models [63]. Furthermore, a bioactive compound from Panax ginseng known as Notoginsenoside R1 (NG)-R1 significantly enhances neuroprotection by upregulating the activity of the estrogen receptor α-dependent PI3K/AKT and Nrf2 pathways and downregulating the NF-κB and MAPK pathways, thereby reducing the expression of inflammatory cytokines and decreasing edema and neuronal cell apoptosis [64]. In addition, a dose of 50 mg/kg of berberine alleviates oxidative stress and ferroptosis by enhancing the Nrf2 pathway and the expression of cellular resilience proteins, including SOD, GSH, GPX4, and SLC7A11, in the brain of triple transgenic AD mouse models [65]. Similarly, berberine (187.75 mg/kg) significantly reduces inflammatory cytokines and brain insulin resistance by increasing GLUT3 expression in neurons of cognitively impaired diabetic rats via inhibition of PI3K/Akt/mTOR signaling [66]. Lastly, oral administration of 300 and 400 mg/kg of polyphenol-rich Boswellia serrata gum extract remarkably suppresses brain insulin resistance and pro-inflammatory cytokines to reverse cognitive decline by enhancing GSH, SOD, and glutamate receptors and inhibiting GSK3β activity in the hippocampus of T2DM rats [67].

### 3.1. Ursolic Acid

Ursolic acid (UA) (3β-hydroxyurs-12-en-28-oic acid) is a naturally occurring pentacyclic triterpenoid found in plants, such as apples, basil, berries, and fruit peels. It exhibits powerful antioxidant, anti-inflammatory, antidiabetic, nephroprotective, and neuroprotective potential, along with its excellent safety and tolerability profile [68]. Recent preclinical evidence demonstrates that dietary intake of UA significantly prevents or attenuates oxidative damage, inflammation [69], and related chronic disorders by targeting the Nrf2 pathway and cellular resilience genes (Figure 3) and proteins in vitro and in vivo (Table 2) [70]. Emerging evidence correlates diabetes with impaired adult neurogenesis, which is crucial for the maintenance of synaptic plasticity and hippocampal functioning, culminating in dementia and AD progression [71]. Accordingly, the proneurogenic effects of the active compounds rosmarinic acid and UA in reversing the deficits in spatial and recognition memory, as well as changes in anxiety induced by Aβ1-42 subtype, have been reported. Specifically, treatment with rosmarinic acid and UA normalized neuronal density and the expression levels of neurogenic (Ki67, NeuN, and DCX) and synaptic (Syn I, II, III, Synaptophysin, and PSD-95) markers [71]. More recently, the same authors revealed the potential neuroprotective effects of UA and rosmarinic acid in comparison to donepezil in AD mouse models. Notably, the combination of these active treatments significantly reduced Aβ plaques and improved brain health (social memory and hippocampal neurogenesis) compared to the Aβ1-42 subtype and donepezil-treated groups, suggesting that UA and rosmarinic acid are potent neuroprotective agents for AD [72]. Furthermore, recent data reveal that UA supplementation significantly enhanced endurance/resistance training and improved spatial memory changes via activation of the Nrf2 pathway and resilience proteins CAT, GPx, and GSH [73]. Moreover, a low dose of UA (5 μM) and acteoside (40 μM) synergistically protected against H_2_O_2_-induced neurotoxicity by the regulation of the AKT/mTOR signaling (Table 2) [74]. Of note, UA prevented Aβ-induced proteotoxic stress, specifically by reducing the amount of Aβ and increasing proteasome activity in *C. elegans* [75]. Intriguingly, the neuroprotective effects of UA and p-coumaric acid (p-CA) from Cornus fructus against Aβ25-35 fragment-induced toxicity have also been observed in PC12 cells. Indeed, p-CA and UA significantly inhibited the expression of iNOS and COX-2 and nuclear translocation of NF-κB, as well as activated the phosphorylation of IκB-α. In particular, UA exclusively reduced ERK1/2, p-38, and JNK phosphorylation, but p-CA suppressed ERK1/2 and JNK phosphorylation [76]. Importantly, molecular docking studies showed that carnosic acid, rosmaric acid, and UA potentially inhibited acetylcholine esterase (AChE) and BACE1, exhibiting binding energies comparable to those of donepezil and therefore could be used to treat AD pathogenesis [77]. An interesting analysis based on machine learning techniques predicted that UA can be considered an effective drug against AD by inhibiting Keap1 and activating Nrf2 to prevent neuronal toxicity caused by Aβ [78]. Moreover, a recent study reported that metabolites from *Eucalyptus tereticornis* leaf extract, including UA, ursolic acid lactone, and oleanolic acid, have powerful anti-inflammatory activity. This activity stems from their ability to downregulate pro-inflammatory gene expression, particularly IL6 and IL1β, chemokines (CXCL3), inflammatory mediators (MMP8 and MMP13), and the JAK-STAT signaling pathway in macrophage cells, suggesting that they may be a promising option for breaking the link between inflammation and insulin resistance [79]. Finally, a synergistic treatment with UA (500 mg) and resistance/endurance training significantly increased T3DM biomarkers, such as brain-derived neurotrophic factor (BDNF) and IGF-1, by reversing the cognitive disorder caused by diabetes in the hippocampus of diabetic aged rats [80]. Overall, the data show that UA impacts brain insulin resistance directly by regulating insulin signaling pathways in brain cells, and indirectly by reducing systemic inflammation and oxidative stress that affect brain function, specifically by targeting the Nrf2 pathway and stress resilience enzymes.

### 3.2. Verbascoside

Verbascoside (VB) is extracted from the herbaceous plant *Verbascum sinuatum* [81]. VB (β-(3′,4′-dihydroxyphenyl)ethyl-O-α-L-rhamnopyranosyl(1→3)-β-D-(4-O-caffeoyl)-glucopyranoside) is a phenylpropanoid glycoside structurally composed of a hydroxytyrosol residue linked to caffeic acid, esterified to a disaccharide (rhamnose linked to glucose) through glycosidic and ester bonds (Table 2) [82]. Also known as acteoside, it is a water-soluble compound widely distributed among various plant species belonging to the order Lamiales (e.g., Lamiaceae, Verbenaceae, Plantaginaceae, Scrophulariaceae, Acanthaceae, Orobanchaceae), to which anti-inflammatory, immunomodulatory, antioxidant, free radical scavenging, neuroprotective, antibacterial, antithrombotic, wound healing, and antitumor activities are attributed (Figure 3) [83,84]. Alterations in the gut microbes/metabolites are involved in the development of cognitive dysfunction. Accordingly, a microbiome-metabolomics analysis study revealed the potential protective effect of VB in alleviating cognitive impairment in db/db mice. Specifically, VB increased the gut microbiota diversity, improved intestinal dysbiosis, attenuated intestinal barrier disruption, reduced the levels of inflammatory factors, regulated the expression of the metabolites associated with cognitive function, and enhanced the central insulin sensitivity and hippocampal synaptogenesis signaling (Table 2) [85]. VB significantly blocked microglia and astrocyte activation in the brain of APP/PS1 mice by suppressing the generation of IL-1β as well as IL-6, and boosting IL-4, IL-10, and TGF-β in vivo, similar to the results obtained in vitro. Furthermore, VB effectively restrained the phosphorylation of IKKα + β, IκBα, and NF-κB-p65 in APP/PS1 mice; LPS-induced BV2 cells, and Aβ-stimulated N2a cells, and lowered the tendency of NF-κB-p65 translocation towards the nucleus in vitro. These results demonstrate that the neuroprotective effect of VB is related to the modulation of neuroinflammation via the NF-κB-p65 pathway, making VB a hopeful candidate drug for the prevention and treatment of AD [86]. Overall, VB inhibits brain insulin resistance both directly and indirectly. It acts directly by scavenging ROS and enhancing the expression of antioxidant enzymes and indirectly through the gut–brain axis, for example, by protecting pancreatic β-cells from stress and inflammation, improving gut microbiota composition, and attenuating brain damage. This dual action offers a promising therapeutic strategy to reduce the risk of developing AD.

### 3.3. Diosmin

Diosmin (3′,5,7-trihydroxy-4′-methoxyflavone-7-rhamnoglucoside) is a dietary flavonoid found in citrus fruits and the leaves of oranges and lemons. It regulates biomarkers of glycemic control, lipid profiles, renal function, and endogenous antioxidant enzymes; modulates signaling pathways related to glucose uptake and insulin sensitivity, blood-lipid-lowering activities, and stress resilience response; and protects capillaries and neuronal cells, mainly by reducing systemic oxidative stress and inflammation (Figure 1) [87]. Recent research reported that a dose of 50 mg/kg of diosmin orally administered for 6 weeks significantly reduced cognitive dysfunction and hippocampal neuronal loss and apoptosis, potentially by upregulating the PI3K/AKT pathway in AD rats in a dose-dependent manner [88]. Moreover, diosmin at doses of 50 and 100 mg/kg administered via the intraperitoneal route significantly restored cognitive functions (working and long-term spatial memory) by enhancing the activity of endogenous antioxidants (e.g., GSH, GPx, SOD, and CAT) in the mitochondrial fraction of the brain and suppressing quinolinic acid-induced mitotoxicity and neurotoxicity in a rat model of neurodegeneration [89]. Interestingly, diosmin reduced cerebral Aβ oligomers, tau hyperphosphorylation, and cognitive impairment by increasing transient receptor potential canonical 6 (TRPC6) and reducing interferone-γ (IFNγ) and pro-inflammatory cytokines (TNFα and IL-12) in a dose-dependent manner. In particular, its major bioactive metabolite diosmetin could be contributing to the anti-AD activities of diosmin in vitro and in 3 × Tg-AD mouse models (Table 2) [90]. Taken together, these results indicate that diosmin and its major metabolite diosmectin inhibit cerebral Aβ levels, tau hyperphosphorylation, and cognitive impairment. These neuroprotective effects are achieved by inhibiting GSK-3α/β and activating PI3K/AKT and Nrf2 pathways, making it a potential new drug candidate for the treatment or prevention of AD.

### 3.4. Tanshinone

Tanshinones are a family of lipophilic diterpenoids extracted from the dried roots of *Salvia miltiorrhiza* Bunge, also known as “Dan-shen.” They are differentiated based on their molecular structure into cryptotanshinone (CT), tanshinone I (Tan I), tanshinone IIA (Tan IIA), and dihydrotanshinone (DT). Tanshinones are used in traditional Chinese medicine for the treatment of cardiovascular and cerebrovascular diseases. Various pharmacological activities have been attributed to tanshinones. Among these, antibacterial, antioxidant, antineoplastic, and antiangiogenic activities stand out (Figure 3) [91,92]. Tan IIA has been shown to be effective in treating TD2M and related neurological disorders, including AD, by activating the Nrf2-signaling pathway and stress resilience genes [93]. Notably, Tan IIA significantly improved cognitive deficits in aged rat models of POCD by suppressing hippocampal inflammation and ferroptosis through reducing MDA and 4-HNE contents, and enhancing SOD activity and GSH levels, primarily targeting the activation of the Nrf2/SLC7A11/GPX4 axis [94]. Of note, a nanodrug delivery system co-loaded with icariin and Tan IIA liposomes was able to pass the BBB and improve AD-like pathological features, including inhibiting neuroinflammation and oxidative stress, reducing apoptosis, protecting neurons, and ultimately enhancing cognitive function by targeting low-density lipoprotein receptor-related protein-1 (LRP1) and Angiopep-2-modified long-circulating (Ang2) in APP/PS1 mice [95]. In vivo, Tan IIA treatment improved neuronal morphology and attenuated oxidative stress and neuroinflammation in the brain tissue of AD mice. In vitro, Tan-IIA showed dose-dependent effects by reversing the Aβ1-42-induced reduction in neural stem cell viability, apoptosis, oxidative stress, and neuroinflammation by modulating the NEAT1/miR-291a-3p/Rab22a/NF-κB signaling pathway [96]. Tan IIA and tetramethylpyrazine O/W composite nanoemulsions inhibited the MAPK/ERK/CREB signaling pathway and effectively alleviated cognitive impairment, oxidative stress injury, and neuronal apoptosis in AD rats [97]. Other evidence indicated that intraperitoneal treatment with TIIA and CT (10 mg/kg) attenuated memory decline in Aβ1-42-injected mice, in a dose-dependent manner by a remarkable reduction in the expression of GFAP, S100β, COX-2, iNOS, and NF-kBp65 after 21 days [98]. Tan IIA-loaded CS nanoparticles significantly prolonged the lifespan and attenuated the AD-like symptoms, including reducing paralysis and the Aβ deposition by inhibiting the oxidative stress and promoting autophagy in *C. elegans* [99]. Tan IIA reduced NFT and the inflammatory response and oxidative stress reaction in the hippocampus of AD rats, by upregulating the expression of CREB, BDNF, and tropomyosin receptor kinase B (TrkB) in the hippocampal tissue of streptozotocin-injured rats (Table 2) [100]. Finally, Tan IIA treatment (15 mg/kg and 30 mg/kg) markedly ameliorated behavioral deficits and improved spatial learning and memory function, attenuated tau hyperphosphorylation and prevented neuronal loss and apoptosis in the parietal cortex and hippocampus, reversed cholinergic dysfunction and reduced oxidative stress in APP/PS1 transgenic mice via the upregulation of the PI3K/Akt/GSK-3β signaling pathway [101].

### 3.5. Baicalein

Baicalein (5,6,7-trihydroxyflavone 7-O-beta-D-glucuronide) (BA) is a flavone extracted from the roots of *Scutellaria baicalensis Georgi* (Labiatae). It has gained interest for its various pharmacological activities, particularly antioxidant, antiviral, anticancer, antidiabetic, anti-inflammatory, cardio-protective, acetyl-cholinesterase-inhibitory, and neuroprotective potential (Figure 3) [102]. Previous literature suggests this bioactive ingredient may help prevent and treat renal and neurological disorders [103,104]. Recent evidence demonstrates that BA mitigates neurotoxic mechanisms and could be a potential complementary nutritional approach for managing neurodegenerative diseases by targeting the Nrf2 pathway and stress resilience enzymes, offering a safer and more holistic alternative to conventional therapies [105]. Compelling evidence reveals that BA permeates the BBB within 20–30 min of administration and reaches the brain to promote neuroprotective effects. Consistent with this concept, BA decreased the Aβ plaque deposition in the brain, attenuated NLRP3 inflammasome activation and neuronal apoptosis by targeting the HMOX1/PDE4D axis in AD rodents [106]. Moreover, BA treatment (10 mg/kg) in combination with memantine (20 mg/kg) significantly reduces oxidative stress, Aβ plaque formation, and increases the expression of BDNF in the Aβ-induced AD model in albino Wistar rats (Table 2) [107]. Similarly, a dose of 200 mg/kg of BA decreases neuroinflammation and increases BDNF expression via inhibition of CX3C receptor 1 (CX3CR1)/NF-κB pathway, thereby improving the learning and memory ability of 3 × Tg-AD mice [108]. Lastly, a recent study performed by Liu and coworkers demonstrated that BA mitigates hepatic and muscular insulin resistance through activation of the PI3K/AKT signal pathway, both in vitro and in vivo. Moreover, it enhances glucose uptake in skeletal muscle cells under insulin resistance conditions through the Ca^2+^/calmodulin-dependent protein kinase II (CaMKII)/AMPK/GLUT4 signaling pathways in a GLP-1R-dependent manner [109].

Collectively, these findings show that BA acts through both direct and indirect mechanisms to mitigate insulin resistance and the risk of AD. It does this by directly inhibiting oxidative stress markers such as hydroxyl radical, while indirectly activating important signaling pathways, specifically PI3K/Akt and Nrf2, to protect cells and promote brain health.

### 3.6. Cynarin

Cynarin is the main polyphenolic compound extracted from artichoke (*Cynara scolymus* L.) that shows excellent anti-inflammatory, anti-aging, anti-glicative, and neuroprotective properties. Emerging evidence highlights that cynarin reduces neuroinflammation, microglial ferroptosis, ROS, and cell death via activation of the Nrf2 antioxidant signaling (Figure 3) [110,111]. This activation increases the expression of antioxidant resilience genes, including SOD and GPx, offering protection to neuronal cells from oxidative stress [112]. Furthermore, artichoke extract has been shown to improve brain damage and memory deficits by inhibiting oxidation and inflammation. Studies suggest that artichoke may serve as a promising alternative therapeutic agent for treating neurological diseases. The beneficial effects of artichoke are attributed to its bioactive phenolic compounds, especially the caffeoylquinic acid derivative, cynarin [112,113,114]. Indeed, the neuroprotective effects of artichoke and its active constituents in attenuating AD have been elucidated. Accordingly, recent findings reported that a high dose of artichoke extract (1.6 g/kg) administered for 14 days effectively ameliorated exposure to diethylnitrosamine-induced brain toxicity by mitigating oxidant parameters and exerting antioxidant and antiapoptotic effects via upregulating the Klotho/PPARγ signaling (Table 2) [112]. The artichoke leaf extracts represent a promising botanical therapeutic approach for managing oxidative stress, neurotrophin secretion, and neuroinflammation, common in neurodegenerative disorders [113]. In mice with streptozotocin-induced sporadic AD, significant improvements in cognitive function and spatial memory recovery, as well as a significant reduction in the inflammatory biomarker TNF-α, Aβ, and tau protein levels, were observed [114]. Moreover, cynarin inhibited the catabolism of nucleus pulposus cells, increased the expression of key ferroptosis-inhibiting genes such as Gpx4 and Nrf2, suppressed the increase in cellular Fe^2+^, lipid peroxides, and ROS dose-dependently both in vitro and in vivo [115]. Finally, a recent report suggested that cynarin, chlorogenic acid, and rosmarinic acid derived from artichoke potentially inhibit matrix metalloproteinase-9 (MMP-9) catalytic site at the picomolar scale [116].

**Table 2:** Summary of the potential molecular pathways upregulated ↑ or downregulated ↓ by functional nutrients in Alzheimer’s disease.

Functional Nutrients	Pathways	Outcomes	Ref.
Ursolic Acid and rosmarinic acid	↑ Syn I, II, III, Synaptophysin, and PSD-95 Ki67, NeuN, and DCX	Reverses the deficits in spatial and recognition memory, as well as changes in anxiety induced by the Aβ1-42 subtype in comparison to donepezil in AD mouse models	[71,72]
Ursolic Acid	↑ Nrf2, CAT, GPx, and GSH	Increases endurance/resistance training and improves spatial memory changes	[73]
Ursolic acid and acteoside	↑ ATG5 and Beclin-1 ↓ AKT/mTOR ↓ caspase-3	Protects against H_2_O_2_-induced nerve damage in AD pathogenesis	[74]
Ursolic acid	↓ Aβ	Prevents Aβ-induced proteotoxic stress in *C. elegans*	[75]
Ursolic acid and p-coumaric acid	↑ IκB-α ↓ NF-κB, iNOS, and COX-2 ↓ ERK1/2, p-38, and JNK	Exerts neuroprotective effects against Aβ25-35 fragment-induced toxicity in PC12 cells	[76]
Ursolic acid plus carnosic acid and rosmaric acid	↓ AChE and BACE1	Docking analysis exhibits binding energies comparable to those of donepezil for the treatment of AD	[77]
Ursolic acid	↑ Nrf2 ↓ Keap1	Machine learning techniques predict that this compound prevents neuronal toxicity caused by Aβ	[78]
Ursolic acid, ursolic acid lactone, and oleanolic acid	↓ IL6 and IL1β ↓ CXCL3 ↓ MMP8 and MMP13 ↓ JAK-STAT	Reduces inflammation and insulin resistance in macrophage cells	[79]
UA and resistance/endurance training	↑ BDNF and IGF-1	Reverses cognitive and memory deficits caused by diabetes in the hippocampus of aged rats	[80]
Verbascoside	↓ IL-1β and IL-6 ↑ IL-4, IL-10, and TGF-β ↑ IκBα ↓ NF-κB-p65	Blocks microglia and astrocyte activation in the brain of APP/PS1 mice and in N2a cells	[85,86]
Diosmin	↑ PI3K/AKT ↓ IL-1β, IL-6, and TNF-α ↓ Bax ↑ Bcl-2	Inhibits neuronal apoptosis and neuroinflammatory responses to improve cognitive dysfunction in AD rats	[87,88]
Diosmin	↑ GSH, GPx, SOD, and CAT	Restores cognitive functions (working and long-term spatial memory) by enhancing the activity of endogenous antioxidants and mitochondrial complex activities in AD rat models	[89]
Diosmin	↑TRPC6 ↓ IFNγ, TNFα, and IL-12 ↓ GSK-3 ↓ γ-secretase ↓ Aβ	Prevents and treats AD and mild cognitive impairment	[90]
Tanshinone IIA	↑ Nrf2, SOD, CAT ↑SLC7A11/GPX4 ↓ MDA and 4-HNE	Improves cognitive deficits in aged rat models by suppressing hippocampal inflammation and ferroptosis	[91,92,93,94]
Nanodrug delivery system by co-loading icariin and Tanshinone IIA liposomes	↑ LRP1 and Ang2 ↑ Bcl-2 and Bcl-XL ↓ Bax, Bad, and Bak	Inhibits AD-like pathological features, including neuroinflammation, oxidative stress, and apoptosis, and enhances cognitive function in APP/PS1 mice	[95]
Tanshinone IIA	↓ NEAT1/miR-291a-3p/Rab22a/NF-κB	Improves neuronal morphology and attenuates Aβ1-42-induced oxidative stress and neuroinflammation in the brain tissue of AD mice	[96]
Tanshinone IIA and tetramethylpyrazine O/W composite nanoemulsions	↓ MAPK/ERK/CREB	Alleviates cognitive impairment, oxidative stress injury, and neuronal apoptosis in AD rats	[97]
Tanshinone IIA and cryptotanshinone	↓ GFAP, S100β, COX-2, iNOS, and NF-kBp65	Attenuates memory decline in Aβ1-42-injected mice in a dose-dependent manner	[98]
Tanshinone IIA-loaded CS nanoparticles	↑ DAF-16/SOD3	Prolongs the lifespan and attenuates paralysis and the Aβ deposition by inhibiting the oxidative stress and promoting autophagy in *C. elegans*	[99]
Tanshinone IIA	↑ CREB, BDNF, TrkB	Reduces neurofibrillary tangles, the inflammatory response, and oxidative stress reaction in the hippocampus of AD rats	[100]
Tanshinone IIA	↑ PI3K/Akt/GSK-3β	Ameliorates behavioral deficits and improves spatial learning and memory function by attenuating tau hyperphosphorylation and preventing neuronal loss and apoptosis in *C. elegans* and in APP/PS1 transgenic mice	[101]
Baicalein	↑ Nrf2 ↑ HMOX1/PDE4D ↓ NLRP3	Inhibits the microglial apoptosis and pro-inflammatory factors and decreases the plaque deposition in the brain of rats	[102,103,104,105,106]
Baicalein plus memantine	↑ BDNF	Reduces oxidative stress, Aβ plaque formation in AD rats	[107]
Baicalein	↑ BDNF ↓ CX3CR1/NF-κB	Decreases neuroinflammation and improves learning and memory ability in 3 × Tg-AD mice	[108]
Baicalein	↑ PI3K/AKT ↑ CaMKII/AMPK/GLUT4	Mitigates hepatic and muscular insulin resistance and enhances glucose uptake by targeting insulin signaling in a GLP-1R-dependent manner in vitro and in vivo	[109]
Cynarin	↑ Nrf2 ↓ NLRP3	Reduces the level of neuroinflammation and microglial ferroptosis in vitro and in mice	[110]
Cynarin	↑ Nrf2/AMPK/SIRT3	Inhibits lipid peroxidation and the transcription of downstream antioxidant pathways	[111]
Cynarin	↑ Klotho/PPARγ	Mitigates oxidant parameters and exerts antioxidant and antiapoptotic effects	[112]
Cynarin	↓ TNF-α, Aβ, and Tau	Improves cognitive function and spatial memory recovery, as well as reduces inflammatory response in AD rats	[113,114]
Cynarin	↑ Gpx4 and Nrf2	Suppresses the increment of cellular Fe^2+^, lipid peroxides, and ROS in vitro and in vivo	[115]
Cynarin	↓ MMP9	Blocks the MMP-9 catalytic site at the picomolar scale	[116]

## 4. Neurotoxicity of MNPs and Redox Resilience Signaling in Alzheimer’s Disease

**Figure 4:**
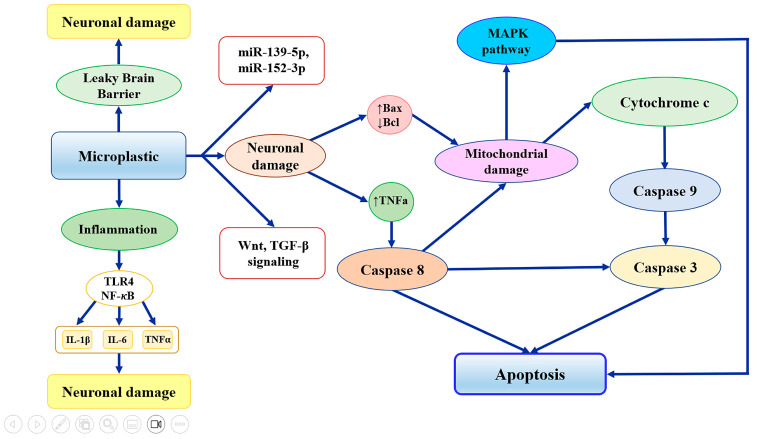
Conceptual scheme of epigenetic modifications induced by microplastics. Activation of various signaling pathways that contribute to upregulating apoptotic processes in neuronal cells. The arrow ↑ is an upregulation; the arrow ↓ is a downregulation.

The neurotoxic effects of MNPs have been primarily studied in aquatic organisms, where they have been linked to behavioral changes, cholinergic dysfunction, and oxidative stress [117]. In mammals, findings are more variable. Some rodent studies report no significant behavioral or cognitive effects following chronic oral exposure [118], while others suggest that NPs may impair cognition, alter neurodevelopment, and reduce neuronal complexity [119,120,121]. Recent evidence has established the dose-dependent nature of NPs’ neurotoxicity, with low-dose NPs either devoid of or exhibiting minimal neurotoxicity compared to high-dose exposure. Accordingly, exposure to high doses of NPs (≥500 µg/d) can significantly affect brain function, causing neurotoxicity and cognitive deficits [121]. Additionally, exposure to 80 nm PS-NPs at a dose of 50 mg for 7 days impaired learning and memory functions in a dose-dependent manner [122]. Emerging evidence suggests that MPs and NPs can cross biological barriers, including the BBB, causing microglia activation and brain damage. This pathological process potentially contributes to AD by disrupting proteins and promoting amyloidosis, although quantifying the exact translocation levels remains challenging due to analytical hurdles [123,124]. Specifically, a concentration of 100 pM of NPs accelerates the nucleation rate of Aβ1-40 and Aβ1-42 subtypes, promoting the formation of more Aβ oligomers and neurotoxicity [123]. The restricted passage through the BBB constrains our understanding of their potential role in neurodegenerative disorders such as AD. Nonetheless, accumulating data indicate that NPs may contribute to neurodegenerative processes and disturb gut–brain axis communication [125,126,127]. In experimental models, NPs from contaminated water have been shown to penetrate the BBB and accumulate in the brain, a property not observed with larger particles [128]. Small PS nanoparticles exhibit neurotoxicity by disrupting enzymatic activity, such as the inhibition of acetylcholinesterase, which plays a crucial role in neuronal function (Figure 4) [129]. Interestingly, Paing and colleagues observed that fluorescent PS-NPs (30–50 nm) orally administered to mice reached the brain and caused problems with memory, but did not affect movement or social behavior. In vitro experiments showed that these particles were mainly taken up by microglia, which then became activated and showed signs of inflammation-related changes in gene expression [127]. This microglial activation negatively affected the activity of nearby neurons, suggesting that brain inflammation caused by microplastics may contribute to cognitive impairment [127]. The neurotoxic effects of NPs appear to be dose- and time-dependent, with lower concentrations and shorter exposures generally exhibiting minimal toxicity. Additionally, particle characteristics play a critical role, as nanoparticles are more readily internalized by cells and exhibit greater toxic potential compared to microparticles [117]. However, due to detection limitations, precise concentrations of NPs in the brain remain unknown [123]. Exposure to MNPs is often associated with oxidative stress and disrupted antioxidant defense, as evidenced by elevated lipid peroxidation and ROS in marine organisms, neuronal cells, and in vitro models [130,131,132]. MPs induce oxidative stress in two primary ways: by raising ROS levels in tissues and cells and by impairing antioxidant enzyme activity, such as SOD, CAT, and GSH, which hinders ROS removal [133,134]. Changes in antioxidant enzyme levels depend on factors such as the size, type, concentration, and exposure duration of MPs, as well as the trophic level of the studied tissues and organisms [135]. Given that oxidative stress is a key contributor to neurodegenerative diseases such as Parkinson’s disease (PD), AD and ALS, plastic particle exposure may represent a risk factor in their development or progression [135]. MPs and NPs trigger oxidative stress that plays a critical role in the progression of AD by promoting tau hyperphosphorylation and the accumulation of toxic pTau oligomers, which ultimately impair neuronal function [136,137]. In line with this, recent evidence reported that a dose of 12.5 mg/kg of PS-NPs induces neuronal cuprotosis, neuronal loss, decreases Nissl body density, impairs synaptic plasticity, and inhibits stress resilience proteins (e.g., GSH, SOD, Nrf2), via activation of the ERK-MAPK pathway, ultimately resulting in learning and memory deficits in murine models [136]. Also, in SH-SY5Y cells, a concentration of 0.75 mg/mL PS-NPs reduced cell viability. These adverse effects were significantly attenuated by treatment with the antioxidant N-acetylcysteine [136]. Likewise, co-exposure to ozone (O_3_) and NPs (12.5 mg/kg) when inhaled together can aggravate BBB damage and cause oxidative stress in the prefrontal cortex, leading to neuroinflammation and neuronal pyroptosis through activation of the p38 MAPK pathway (Figure 4) [137]. Moreover, administration of N-Acetylcysteine (2 g/L) in aqueous solution to mice over a 30-day exposure period can markedly alleviate neuroinflammation and neuronal pyroptosis in the prefrontal cortex, and it reversed the cognitive deficits and anxiety-like behaviors observed in the co-exposed mice [137]. Thus, NPs induce neuroinflammation through mechanisms such as microglial pyroptosis, exacerbating cognitive decline in AD as observed by several studies [136,137,138]. Low doses of NPs, especially those with hydrophobic surfaces, can promote amyloid aggregation and increase oligomeric forms, resulting in cellular damage through elevated ROS and calcium levels [123]. Furthermore, an interesting study found that the synergistic effects of NPs and organic contaminants (pyrene, bisphenol A, 2,2′,4,4′-tetrabromodiphenyl ether, 4,4′-dihydroxydiphenylmethane, or 4-nonylphenol) promote neurotoxicity, insulin aggregation, and the accumulation of Aβ fibrils [139]. In addition, a recent study conducted on a cohort of postmortem human tissues observed the highest concentrations of MNPs in the thyroid (40.4 MP/g), followed by the brain and kidneys, compared to other organs [140]. Finally, a clinical study investigated the potential association between cerebrospinal fluid (CSF), MPs, and the onset of AD. Specifically, increased levels of MPs in the CSF are associated with Aβ deposition and cognitive decline among AD individuals [141]. Overall, recent findings highlight that MNPs may play a significant role in promoting neurotoxicity leading to BBB dysfunction, neuroinflammation, and Aβ aggregation, ultimately resulting in the onset and progression of AD with a size- and dose-dependent action. Currently, there are very few clinical and post-mortem studies in the literature regarding MNPs and AD.

## 5. Functional Nutrients Prevent or Attenuate MNP-Induced Toxicity and the Risk of Chronic Diseases

**Figure 5:**
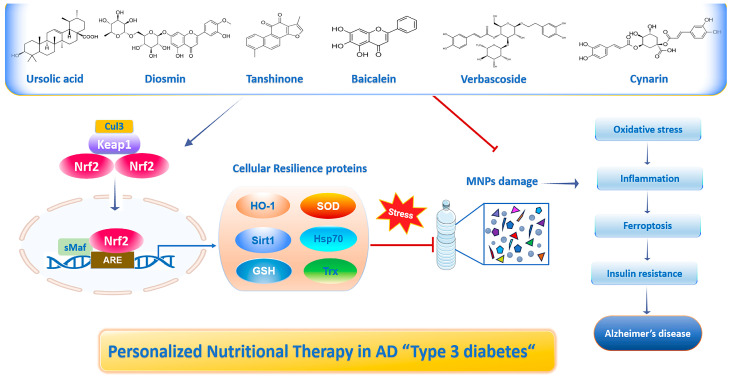
Overview of the potential protective effects of functional nutrients targeting the Nrf2 pathway to counteract MNP damage in neuronal cells.

MNPs, due to their small size, can cross biological barriers and accumulate in cells, tissues, and organs, inducing cellular and molecular changes, which result in toxic effects [142]. Notably, long-term and high-dose exposure to 100 μg/L PS-NPs for 30 days resulted in accumulation in the gut and brain, which promoted chronic inflammation, intestinal permeability, and neurological problems [143]. Given the toxic effects induced by MNPs and related chronic diseases, the question of how to reduce the entry of these MNPs or detoxify them from the human body, particularly the CNS, is a crucial aspect that deserves greater attention (Figure 5). From this new perspective, functional nutrients and/or dietary supplements enriched with bioactive compounds represent a promising strategy to mitigate cellular and neuronal damage and maintain redox balance in the context of environmental pollutants (Table 3).

### 5.1. Tannic Acid and Glycyrrhizic Acid

Recent evidence has shown that plant secondary metabolites, specifically tannic acid and glycyrrhizic acid at a low dose of 0.1 g/L, attenuated ROS and toxicity caused by PE-MPs (1 g/L) by reducing the expression of the glutathione S-transferase-4 gene (*gst-4*) in *C. elegans* [144].

### 5.2. Resveratrol

Another recent study reported that long-term and low-dose oral administration of PS-NPs (15 mg/kg) induced plasma glucose metabolism disorder by increasing the levels of MDA and ROS and NF-κB/MAPK pathway, as well as reducing the activities of antioxidant enzymes like SOD and GSH. The dose of 100 mg/kg of resveratrol mitigated the PS-NP-induced oxidative stress and inflammatory response, exerting a protective effect in mice [145].

### 5.3. Naringin

Similarly, evidence has shown the therapeutic potential of the flavonoid naringin in mitigating the adverse effects of MPs exposure in the endocrine system. Specifically, co-administration of naringin (100 mg/kg) via oral gavage in MPs (1.5 mg/kg) exposed mice significantly ameliorated the alterations in Kallikrein-3 levels, hormone disturbances, and oxidative stress markers by enhancing GSH, SOD, and CAT and inhibiting MDA, thereby preventing ROS production, inflammation, and cellular toxicity [146].

### 5.4. Quercetin

Moreover, PS-NPs accumulate within the intestine, resulting in impairments to intestinal tissue and barrier function, as well as disturbing the expression of immune-response small intestinal genes and gut microbiota composition. In this regard, a multi-omics analysis conducted by Zhao et al. reported that quercetin at a dose of 50 mg/kg alleviated PS-NPs-induced intestinal damage (50 mg/kg) and immune disorders by reversing intestinal flora dysbiosis, and targeting key intestinal genes including *Fam126b*, *Prr7*, *Ggn*, and *Atp11C* in mice [147].

### 5.5. Cyanidin-3-O-Glucoside

Likewise, a dose of 150 mg/kg per day of bayberry-derived anthocyanin cyanidin-3-O-glucoside (C3G) reduced colonic PS-MPs (5 μm in size and 1 mg/mL concentration) accumulation and modulated the gut microbial metabolites in C57BL/6 mice [148]. Intriguingly, C3G treatment significantly changed bacterial gene abundances about phenylalanine, tyrosine, purine, and tryptophan metabolism and related metabolic pathways. In particular, the study observed that C3G upregulated tryptophan metabolites such as indole-3-pyruvate, indole-3-acetamide, and N-acetylserotonin to improve the oxidative damage and intestinal toxicity of PS-MPs to the host by enhancing the degradation and detoxification of PS xenobiotics in colon tissue and feces of mice [148]. Similarly, a concentration of 50 μg/mL of C3G promoted stress resistance and lifespan extension by upregulating DAF-16 expression and its downstream antioxidant genes (*clt-2*, *hsp-16.1*, *sod-3*, *sod-5*) in *C. elegans* exposed to PS-NPs [149].

### 5.6. Nobiletin

Recent research by Yu and colleagues demonstrated that nobiletin, a flavonoid derived from citrus peel, modulates autophagy and mitigates NP-induced toxicity in human intestinal Caco-2 cells [150]. Specifically, nobiletin at doses ranging from 12.5, 25, to 50 μM blocked NP-(100 μg/mL) induced toxicity after 24 h by promoting the nuclear translocation of TFEB mediated by the activation of AMPK and inhibition of mTOR signaling. Furthermore, nobiletin appears to induce the formation of autophagosomes and lysosomes, enhancing the cellular ability to degrade and recycle damaged cells induced by NPs [150]. Therefore, nobiletin holds promise as a therapeutic nutritional agent for mitigating the toxic effects of NPs.

### 5.7. Luteolin

Of note, PS-NPs can trigger oxidative dysregulation, Ca^2+^ imbalance, and iron accumulation, leading to ferroptosis and neuroinflammation. Accordingly, a recent study performed by Tan and coworkers showed that luteolin blocks PS-NPs exposure-induced striatal injury characterized by neuronal degeneration and mitochondrial dysfunction in vitro and in vivo. Notably, a dose of 3 μM of luteolin markedly reduced PS-NPs-induced neurotoxicity (100 nm, 5 mg/kg) by mitigating pro-inflammatory cytokine levels (e.g., IL-1β, IL-6, and TNF-α), enhancing antioxidant resilience proteins (e.g., CAT and SOD), and suppressing the accumulation of lipid peroxidation markers (e.g., MDA), thus alleviating striatal ferroptosis and neurodegeneration via activation of the G6PD/GSH axis and inhibition of the Piezo1/CaN/NFAT1 axis in primary hippocampal neurons and hippocampal tissues of mice [151].

### 5.8. Tamarixetin

Interestingly, the flavonoid tamarixetin significantly recovered PS-MPs-induced hepatic damage by upregulating antioxidant resilience signaling, such as GST, SOD, HO-1, GSR, GPx, CAT, and GSH content, whereas downregulating ROS and MDA, as well as the inflammatory indices, such as IL-1β, NF-κB, IL-6, TNF-α levels, and COX-2 activity in rats [152].

### 5.9. Anthocyanins

A recent study suggested that long-term, low-concentration (0.01 mg L^−1^) PS-MPs exposure disrupted the redox homeostasis, induced oxidative damage, and exacerbated cell apoptosis in planarians, likely due to altered neural gene expressions compared to short-term, high-concentration (83 mg L^−1^) exposure. Surprisingly, a concentration of 20 mg L^−1^ of anthocyanins alleviated these toxic effects [153].

### 5.10. Ginkgetin

Equally important, the bioflavonoid ginkgetin at a dose of 25 mg/kg has shown therapeutic potential against PS-MPs at a dose of 0.01 mg/kg^−1^. Specifically, the study observed that ginkgetin mitigated the PS-MP-induced testicular toxicity by reducing oxidative stress markers, inflammatory cytokines, and apoptotic factors and enhancing antioxidant enzymes in albino rats [154].

### 5.11. Kaempferide

Co-administration of the flavonoid kaempferide (20 mg/kg^−1^) along with PE-MPs (1.5 mg/kg^−1^) significantly mitigated reproductive toxicity by restoring the activities of Nrf2 and related antioxidant enzymes and decreasing the levels of oxidative, inflammatory, and apoptotic markers in male rats [155].

### 5.12. Catechins

Recent evidence indicated that catechin, epicatechin, gallocatechin, and epigallocatechin can reduce oxidative stress in human intestinal epithelial cells and inhibit size-dependent and dose-dependent cytotoxicity of MNPs [156].

### 5.13. Docosahexaenoic Acid-Enriched Phosphatidylserine

An important study reported that PS-NPs (size 100 nm and dose 25 mg/kg) disrupt the hepatic Sirt1-AMPK pathway by suppressing the expression of Sirt1, AMPKα, and PPARα, while increasing the expression of SREBP-1c and TLR4/NF-κB pathway as well as impairing tight junction proteins, ultimately leading to disordered hepatic lipid metabolism. In this regard, administration of 50 mg/kg of the dietary supplement docosahexaenoic acid-enriched phosphatidylserine (DHA-PS) effectively alleviated all this damage in a murine model of liver injury [157].

### 5.14. Functional Food Camellia Pollen

Camellia pollen treatment at a moderate dose of 50 mg significantly attenuated neurotoxicity and neuronal apoptosis mediated by the p53/Bax/Bcl-2 axis in vivo after 15 weeks [158]. Also, in vitro, camellia pollen treatment at a concentration of 40 μg/mL reversed the reduction in cell viability caused by amino-modified PS-NPs and increased the expression of occludin and ZO-1 via the inhibition of the TLR2/MMP9 axis [158]. Lastly, the same study showed that camellia pollen treatment significantly suppressed NPs-induced neuronal injury by reducing GAPDH/Ac-Tau signaling pathway and enhancing Sirt-1 pathway in mouse hippocampal neuron cells (Table 3). Currently, very few studies have investigated the mechanisms of action of functional nutrition and/or nutraceuticals in preventing or suppressing MP-induced cell and tissue toxicity and damage by targeting multiple molecular pathways, including Nrf2 and stress resilience proteins. It is hoped that in the future, the role of nutrients will gain greater relevance in both the prevention and management of MNP-induced systemic toxicity with the goal of minimizing oxidative stress and inflammation as well as apoptosis, and ultimately the risk of chronic diseases in humans.

**Table 3:** Potential mechanisms and pathways upregulated ↑ or downregulated ↓ by functional nutrients to prevent or reverse MNP-induced damage and toxicity, and the risk of chronic diseases.

Functional Nutrients	Pathways	MPs and NPs Damage	Ref.
Tannic acid and glycyrrhizic acid	↓ *gst-4*	Attenuates oxidative damage caused by the dose of 0.1 g/L and 1 g/L of polyethylene (PE)-MPs in *C. elegans*	[144]
Resveratrol	↑ Nrf2, SOD, and GSH ↓ ROS, MDA, MAPK, and NF-κB	Mitigates the PS-NPs-induced glucose and lipid metabolic disorders associated with oxidative stress and inflammatory response in mice	[145]
Naringin	↑ GSH, SOD, and CAT ↓ MAD and ROS	Inhibits the adverse effects of MPs’ exposure in the endocrine system in mice	[146]
Quercetin	↑ Fam126b, Prr7, Ggn, and Atp11C	Alleviates PS-NPs-induced intestinal damage and immune disorders by reversing intestinal flora dysbiosis in rats	[147]
Cyanidin-3-O-glucoside	↑ indole-3-pyruvate, indole-3-acetamide and N-acetylserotonin	Improves oxidative damage and intestinal toxicity of PS-MPs to the host by enhancing the degradation and detoxification of PS xenobiotics in colon tissue and feces of mice	[148]
Cyanidin-3-O-glucoside	↑ DAF-16 ↑ *clt-2*, *hsp-16.1*, *sod-3*, *sod-5*	Promotes stress tolerance and lifespan extension in *C. elegans* exposed to a dose of 100 μg/mL PS-NPs.	[149]
Nobiletin	↑ TFEB and AMPK ↓ mTOR	Induces the formation of autophagosomes and lysosomes, enhancing the cellular ability to degrade and recycle damaged cells induced by the dose of 100 μg/mL of NPs	[150]
Luteolin	↑ CAT and SOD ↑ G6PD/GSH ↓ Piezo1/CaN/NFAT1 ↓ MDA	Reduces PS-NPs-induced neurotoxicity by mitigating oxidative stress, pro-inflammatory cytokines, and ferroptosis in vitro and vivo	[151]
Tamarixetin	↑ GST, SOD, HO-1, GSR, GPx, CAT, and GSH ↓ ROS and MDA ↓ IL-1β, NF-κB, IL-6, TNF-α, and COX-2	Restores PS-MPs-induced hepatic damage by reducing oxidative stress and inflammatory mediators in rats	[152]
Anthocyanins	↑ SOD, CAT, and GPX ↓ MDA ↑ *Djpc2* and *DjFoxG* ↓ *Djcaspase3* and *Djp53*	Alleviates oxidative stress, apoptosis, and neurotoxicity of long-term low concentration of PS-MP (0.01 mg) exposure in planarians	[153]
Ginkgetin	↑ SOD, GSR, GPx, CAT ↑ Bcl-2 ↓ IL-1β, IL-6, TNF-α ↓COX2 ↓ ROS and MDA	Reduces PS-MP-induced testicular toxicity, oxidative stress, inflammation, and apoptosis in albino rats	[154]
Kaempferide	↑ Nrf2, SOD, GPx, CAT, GST, GSR, and HO-1 ↓ ROS and MDA, ↓ NF-κB, IL-1β, IL-6, TNF-α ↓Bax and Caspase-3 ↑ Bcl-2	Mitigates PS-MP-induced reproductive toxicity in male rats via antioxidant, anti-inflammatory, and anti-apoptotic effects	[155]
Catechin, epicatechin, gallocatechin, and epigallocatechin	Not specified	Reduces oxidative stress and inhibits size-dependent (3 μm, 0.3 μm, 80 nm, and 20 nm) and dose-dependent (0.1, 0.01, 10, and 100 μg mL^−1^) cytotoxicity of MPs and NPs in human colon carcinoma (Caco-2) cells	[156]
DHA-PS	↑ Sirt1, AMPKα, and PPARα ↑ ZO-1, occludin, and claudin-1 ↓ SREBP-1c ↓ TLR4/NF-κB	Attenuates oxidative stress, inflammation, and impairs tight junctions in a murine model exposed to PS-NPs-induced hepatotoxicity	[157]
Camellia pollen	↓ TLR2/MMP9 ↓ GAPDH/Ac-Tau ↑ Sirt1 ↓ p53/Bax, ↑ Bcl-2	Alleviates BBB damage, neuronal apoptosis, and AD-like neurotoxicity induced by amino-modified PS-NPs exposure in vitro and in vivo	[158]

## 6. Epigenetic Modulators of the *NFE2L2* Gene

Epigenetic modifications influence gene transcription by altering chromatin structure. This allows for the control of cellular functions in living organisms. Some studies highlight that MPs dysregulate key molecular signaling pathways and can induce significant epigenetic changes, which could be involved in the onset of various pathological disorders (Table 4) [159,160]. The emerging epigenetic impact of MNP exposure is characterized by alterations in chromatin remodeling and miRNA modulation, underscoring their potential to modify the epigenome. *C. elegans* is an animal model with high sensitivity to various toxicants and has been used for detecting the neurotoxicity of MNPs. Compelling studies have examined epigenetic responses, including Wnt signaling and TGF-β signaling (Figure 4), using in vitro and in vivo models such as *C. elegans*, mammalian models (*Daphnia magna* and mice), and human cell lines after MNP exposure [161,162]. Analogous pathways and thus their human counterparts have been predicted and successively identified using an in silico approach (database and algorithms). In particular, the KSR-ERK-MAPK pathway, the FOXO-insulin cascade, and GPX3-HIF-α in humans, all of which are miRNA-mediated pathways, may be affected by MNP exposure. This influence can lead to disruption of key metabolic pathways, apoptosis, cell proliferation, and angiogenesis [163]. Recent research in animal models and cell lines has highlighted the epigenetic impacts associated with MNP exposure, particularly chromatin remodeling and miRNA modulation. Experiments in mice have provided deeper insights into human epigenomic responses. In particular, brain exposure of mice chronically exposed to various doses showed that MPs impaired cognitive function. These effects resulted from significant functional dysregulation of 29 miRNAs, with overexpression of miR-139-5p and miR-152-3p (Figure 4) [164]. These findings may be related to the decline in synaptic function, which leads to PD-induced nerve injury and cognitive decline [165]. Other studies have shown that miR-139-5p plays a role in regulating spatial and working memory through the PI3K/AKT signaling pathway [166]. Furthermore, miR-152-3p activation targeting DNMT1 leads to the loss of cortical interneurons, contributing to a significant decline in synaptic integration [167]. Liang et al. confirmed that MNPs (50 nm) can induce neurodegeneration similar to that observed in AD and PD, primarily by disrupting metabolic activities in neurons of C57BL/6 mice. This disruption is characterized by a decrease in ATP concentration within cells and the concomitant downregulation of ATP-related genes and proteins, indicating the involvement of specific epigenetic mechanisms [168]. Epigenetic modifications are reversible and can influence the expression, activity, and stability of the *NFE2L2* gene [169]. These different forms of epigenetic modifications interact with each other to coordinate the regulation of gene expression, playing an important role in biological processes, such as cellular differentiation, development, disease progression, and stress responses [170]. Recent studies have revealed that plant-based dietary compounds can influence Nrf2 expression through these epigenetic modifications [171]. Chromatin remodeling directly affects DNA accessibility for transcription factors and regulatory proteins in the regulation of gene expression. This mechanism may also play a significant role in the progression of oxidative stress-related diseases by impacting the expression levels of Nrf2 target genes. Methylation of CpG islands in the *Nrf2* gene promoter acts as a repressive mechanism, suppressing Nrf2 transcriptional activity. This results in a reduction in *NFE2L2* gene expression levels [169], consequently compromising the cell’s antioxidant functions [172]. In particular, dietary compounds have been observed to act as inhibitors of DNA methyltransferases (DNMTs), which are considered epigenetic enzymes. This action leads to a reduction in hypermethylation of CpG islands present in the region of the promoter of the *NFE2L2* gene. The result is an increase in Nrf2 protein levels [169], with protective and beneficial effects for oxidative stress-related diseases [173]. DNA demethylation is a process that consists of the elimination of the methyl functional groups from cytosines in DNA by demethylases, enzymes known as TET. This demethylation in the promoter area of the *NFE2L2* gene can activate protein expression levels. In this area of the promoter, the reduction in methylation facilitates the action of transcription factors, which bind regulatory proteins to the promoter, increasing Nrf2 transcription and expression levels, allowing cells to resist oxidative stress, potentially improving the progression of some diseases and their prognosis [172]. Cellular function is modulated by Noncoding RNA regulation, through which noncoding RNAs modulate gene expression and influence gene activity through small interfering RNAs (siRNAs), long noncoding RNAs (lncRNAs), miRNAs, and circular RNAs (circRNAs). These noncoding RNAs modulate the activity of Nrf2 [174], influencing cellular responses to redox state and other pathophysiological processes [175]. Recent studies have revealed that *NFE2L2* gene expression is inhibited by long noncoding RNA through epigenetic processes, facilitating inflammasome activation, for example, in mice and microglial cell models of PD, resulting in neuroinflammation [176].

**Table 4:** Dysregulation of molecular signaling pathways and epigenetic changes induced by microplastics and their potential involvement in the development of biological effects.

Molecular Target/Epigenetic Factors	Type of Epigenetic Alteration	Biological Effects	Ref.
Chromatin structure	Chromatin remodeling induced by MNP exposure	Altered gene transcription and dysregulation of cellular functions	[159,160]
miRNAs	Dysregulation and modulation after MNP exposure	Altered gene transcription and dysregulation of cellular functions	[160]
Wnt	Epigenetic modulation after MNP exposure	Altered cellular proliferation and differentiation	[161]
TGF-β	Epigenetic modulation after MNP exposure	Dysregulation of cell growth and apoptosis	[162]
KSR–ERK–MAPK pathway	miRNA-mediated epigenetic alteration after MNP exposure	Altered metabolism, apoptosis, and proliferation	[163]
FOXO–insulin cascade	miRNA-mediated epigenetic alteration	Impaired metabolic homeostasis	[163]
GPX3–HIF-α pathway	miRNA-mediated epigenetic alteration	Altered angiogenesis and oxidative stress response	[163]
miR-139-5p	Overexpression after chronic MP exposure	Cognitive impairment and synaptic dysfunction	[164]
miR-152-3p	Overexpression after chronic MP exposure	Cognitive decline and loss of synaptic integration	[165]
PI3K/AKT signaling	Regulation by miR-139-5p	Altered spatial and working memory	[166]
DNMT1	Targeted by miR-152-3p	Loss of cortical interneurons, impaired synaptic integration	[167]
ATP and ATP-related genes	Downregulation after MNP exposure	Reduced cellular energy and neurodegeneration	[168]
*NFE2L2* gene	Epigenetic regulation (methylation, demethylation, ncRNA control)	Altered antioxidant defense and stress resilience response	[169,170]
CpG islands in the Nrf2 promoter	Hypermethylation	Suppression of Nrf2 transcription and reduced antioxidant capacity	[171,172]
DNMTs	Inhibition by dietary compounds	Reduced Nrf2 promoter hypermethylation and increased Nrf2 protein	[169,173]
TET demethylases	DNA demethylation of the Nrf2 promoter	Increased Nrf2 transcription, enhanced resistance to oxidative stress	[172]
siRNA, lncRNA, miRNA, circRNA	Epigenetic modulation of Nrf2 activity	Regulation of redox state and pathophysiological processes	[174]
lncRNA	Epigenetic inhibition of Nrf2	Inflammasome activation and neuroinflammation in PD models	[175]
Synaptic function	miRNA dysregulation after MP exposure	Synaptic decline leading to cognitive impairment and PD-like injury	[176]

### 6.1. Micro- and Nanoplastics Induce Ferroptosis Targeting the NFE2L2 Gene

Ferroptosis, an iron-dependent form of programmed cell death characterized by lipid peroxidation and oxidative stress, is increasingly recognized as a key destructive process in Alzheimer’s disease (AD) pathogenesis due to shared features like iron dysregulation [177]. Recent evidence suggests that inhibiting MNP-induced ferroptosis, particularly by targeting the NFE2L2 (encoding Nrf2) gene, holds promise as an anti-AD therapeutic strategy [177,178]. Emerging studies report that MPs inhibit the Keap1-Nrf2 pathway and its downstream genes, induce lipid peroxidation and ferroptosis, ultimately causing toxicity and damage to structures and functions (Table 5) [179,180,181,182,183]. Consistent with these findings, Lan and colleagues found that co-exposure of PS-MPs with a diameter of 1 μm and a dose of 10 mg/L in synergy with cadmium (10 mg/L) inhibits the NFE2L2 gene by inducing ferroptosis and barrier toxicity in vivo [179]. In the subsequent toxicological experiments, Qui et al. demonstrated that PS-NPs (300 nm size and 1 mg/kg dose) co-exposure with cadmium (1.5 mg/kg) significantly increased the MDA levels and 4-HNE and 8-OHDG expressions, while decreasing the activity of the Nrf2 pathway and stress resilience genes. More importantly, these outcomes also suggest that cadmium and PS-NPs co-exposure synergistically increased iron concentration and induced ferroptosis More importantly, these outcomes also suggest that cadmium and PS-NPs co-exposure synergistically increased iron concentration and induced ferroptosis by downregulating the expression of antioxidant pathways, i.e., Nrf2, HO-1, and NQO1 and antiferroptotic proteins, i.e., cystine-glutamate antiporter (SLC7A11), glutathione peroxidase 4 (GPX4), ferritin heavy chain 1 (FTH1), and upregulating 8-hydroxy-2′-deoxyguanosine (8-OHDG) and 4-HNE, as well as ferritin light chain (FTL), prostaglandin-endoperoxide synthase 2 (PTGS2), transferrin receptor 1 (TFR), ferritin light chain (FTL), and high-mobility group box 1 (HMGB1) in vivo [180]. Furthermore, a study performed by Yin and coworkers demonstrated that MPs can affect brain tissue by destroying the BBB and increasing glutamine and glutamate synthesis via the inhibition of the Nrf2-Keap1-HO-1/NQO1 signaling pathway (Table 5), ultimately triggering autophagy-dependent ferroptosis through reduction in GPX protein expression in the chicken cerebellum [181]. Interestingly, a study conducted by Liang et al. observed that high concentrations of PS-NPs induced toxicity and ferroptosis in intestinal epithelial cells and in intestinal epithelial-specific Nrf2-deficient mice [182]. Additionally, a high-fat diet further exacerbated this effect, suggesting that individuals with reduced Nrf2 activity and poor dietary habits may be particularly vulnerable to PS-NP-induced intestinal damage [182]. Moreover, a recent study by Fu et al. reported that PS-NPs (50 and 90 nm in diameter, respectively) at a concentration of 12.5 and 25 μg/mL can pass the blood–testis barrier and induce ferroptosis through a significant reduction in *NFE2L2* and *GPX4* gene expression and an increase in the levels of lipid peroxidant marker MDA both in vitro and in vivo. The same authors also showed that ferrostatin 1, a ferroptosis inhibitor, inhibited PS-NPs-induced increased intracellular free divalent iron levels, high MDA levels, and low GSH levels. These results suggest that PS-NPs accelerate ferroptosis when *NFE2L2* gene function and its target genes FPN1, GPX4, and HO-1 are blocked, indicating that Nrf2 plays a protective role in PS-NPs-induced ferroptosis [183]. Finally, a study performed by Shi et al. found that long-term exposure to high-dose PS-MPs promotes cytotoxicity and ferroptosis in vitro and in vivo [184]. Specifically, a high dose of 25 μg/mL in human hepatocyte cells and 10 mg/L in oral drinking water in mice increases liver cell senescence mediated by ferroptosis targeting HO-1/Nrf2 pathway, respectively [184]. Taken together, the data demonstrate that MNPs induce toxicity and ferroptosis in a dose-dependent manner by inhibiting the expression of the *NFE2L2* gene and downstream resilience proteins, ultimately contributing to the development of many chronic disorders.

**Table 5:** MNPs promote ferroptosis by upregulating ↑ or downregulating ↓ molecular pathways and stress resilience genes.

MNPs’ Dose and Size	Pathways	Outcomes	Ref.
1 μm and dose of 10 mg/L PS-MPs and cadmium	↓ Nrf2	Induce ferroptosis and barrier toxicity in vivo	[178]
300 nm and dose 1 mg/kg PS-NPs and cadmium	↑ MDA, 4-HNE, 8-OHDG ↓ Nrf2, SLC7A11, GPX4, PTGS2, HMGB1, FTH1 and FTL	Increase oxidative stress, ferroptosis, and excessive mitophagy ultimately aggravating kidney damage in mice	[179]
5 μm and 10 mg/L and 100 mg/L PS-MPs	↓Nrf2/HO1/NQO1 ↓ GPX	Promote autophagy-dependent ferroptosis and apoptosis in cerebellar tissue of chickens via the liver-brain axis	[180]
50 nm and dose of 0.25, 2.5, 25, and 250 mg/kg PS-NPs	↓ Nrf2, HO-1, GPX4, SLC7A11, FTH1 ↑ 8-OHDG, 4-HNE	Induce toxicity and ferroptosis in intestinal epithelial cells and in intestine-specific Nrf2 knockout mice in a dose-dependent manner	[181]
50 and 90 nm and dose of 12.5 and 25 μg/mL PS-NPs	↓ Nrf2 and GPX4, FPN1 and HO-1 ↑ MDA	Cross blood–testis barrier and induce ferroptosis in vitro and in vivo	[182]
5 μm and dose of 25 μg/mL PS-MPs	↓ Nrf2/HO-1 ↓ GPX4	Increase liver cell senescence mediated by ferroptosis targeting antioxidant pathways	[183]

### 6.2. Functional Nutrients as Epigenetic Modulators of the NFE2L2 Gene

Consuming a diet with an adequate amount of fruits and vegetables meets nutritional needs and also increases defense capacity against inflammation and oxidative stress-associated diseases. Some nutrients have been reported to have antioxidant potential in in vitro and in vivo experimental research because they can directly scavenge ROS and enhance the expression of cellular antioxidant enzymes, preventing cellular damage mediated by oxidative stress [185]. Oxidative stress is implicated in the induction of several acute and chronic diseases, including neurodegenerative diseases with consequent kidney damage. These bioactive dietary nutrients effectively act as modulators of the *NFE2L2* gene through epigenetic alterations, such as histone modifications, DNA methylation, and miRNA alterations [169]. The human diet is rich in a wide variety of nutrients with health-beneficial effects. These nutrients, containing bioactive compounds present in the diet, can influence the Nrf2 pathway. Importantly, the Nrf2 signaling network has been considered a promising target against oxidative stress-mediated damage, such as neurotoxicity [185]. These naturally occurring compounds can modulate the Keap1-Nrf2 pathway. In this review, we discuss the role of the Nrf2 signaling pathway in neurodegenerative diseases. The administration of some compounds, i.e., ursolic acid [186], and tanshinone is involved in epigenetic modulation of the Nrf2 pathway [187]. A particularly promising area of research concerns the identification and application of bioactive nutrients as effective modulators of the Nrf2 pathway. These natural compounds exert significant anti-inflammatory and antioxidant effects, and their capacity to influence Nrf2 through epigenetic modifications is increasingly evident [188]. These therapeutic effects are often attributed to their ability to regulate the Nrf2-signaling pathway [189], enhancing the anti-inflammatory, antioxidant, and anti-apoptosis defenses of the organism [190]. The growing depth to clarify the molecular mechanisms of Nrf2, including its regulators and its complex role, is opening new opportunities for the prevention and treatment of several diseases. The reversible nature of epigenetic alterations makes their modulation a particularly attractive strategy for therapeutic intervention, especially with the use of bioactive nutrients that can act on multiple levels (transcriptional, post-transcriptional, and post-translational) [191]. Ongoing research into how bioactive nutrients influence the human epigenome and the Nrf2 pathway is crucial. Although several checkpoints in the complex Nrf2 pathway have been clarified, and bioactive nutrients, such as ursolic acid [186] and tanshinone [187], are already known to be epigenetic modifiers of Nrf2, there is still much to explore.

*Ursolic acid*, present in blueberries, apples, basil, and rosemary, is a pentacyclic triterpenoid that has been studied for its capacity to epigenetically regulate Nrf2 [186]. In vitro research on PC-3 and LNCaP cell lines found that ursolic acid was able to induce elevated expression of SETD7, a protein methyltransferase; furthermore, subsequent gene silencing experiments demonstrated reduced levels of Nrf2 expression; therefore, methylation of the Nrf2 protein by SETD7 is relevant in these mechanisms [192]. Another study showed that ursolic acid activated the Nrf2 via demethylation of the *NFE2L2* gene in the promoter area. This process was accompanied by a reduction in DNMTs and HDACS (Table 6) [186]. A deeper understanding of the processes regulating the *NFE2L2* gene could provide new targets and strategies for treating related diseases. Identifying the interactions between their different alterations in the physio-pathological state would allow us to clarify the mechanisms of Nrf2 regulation and the development of new therapeutic strategies.

*Tanshinone*, a fat-soluble extract from the medicinal plant Salvia miltiorrhiza Burge, is known for its cytoprotective effects. Tanshinone has been explored as a compound that enhances the transcriptional mechanism of Nrf2 activation at the promoters responsible for the production of target molecules [187]. Tanshinone can induce Nrf2 expression through epigenetic processes, as it has been confirmed in in vitro and in vivo models of rifampicin-induced liver injury. Research revealed that Tanshinone IIA causes demethylation in the promoter of the *NFE2L2* gene and is implicated in the induction of Nrf2 messenger and protein, a process guaranteed by the presence of the enzyme TET2. The study did not detect significant changes in DNMTs but found elevated production of demethylases of DNA in human hepatocytes and HepaRG cells, which prevented rifampicin-induced liver damage [187]. Another study reported a reduction in the DNMTs, Hdac1, Hdac3, and Hdac8 in JB6 P+ cells (Table 6) [193].

**Table 6:** Effects of functional nutrients targeting molecular pathways involved in epigenetic regulation of the *NFE2L2* gene. The arrow ↑ is an upregulation; the arrow ↓ is a downregulation.

Nutrient Epigenetic Modulator	Epigenetic Regulation of *NFE2L2* Gene	Signaling Pathway/Molecular Targets	Outcomes/Effects	Ref.
Ursolic acid	Methylation of the Nrf2 protein Demethylation of the *NFE2L2* gene promoter	↑ SETD7 ↓ DNMT, ↓ HADCs	Increased defense capacity against inflammation and oxidative stress; prevention of cellular damage; potential neuroprotection	[187,193]
Tanshinone	Demethylation of the *NFE2L2* gene promoter	↑ TET2 ↓ DNMT, ↓ HADCs	Cytoprotective effects; enhancement of Nrf2 transcriptional machinery	[188,194]

### 6.3. The Role of Nrf2 in Neurological Disorders

**Figure 6:**
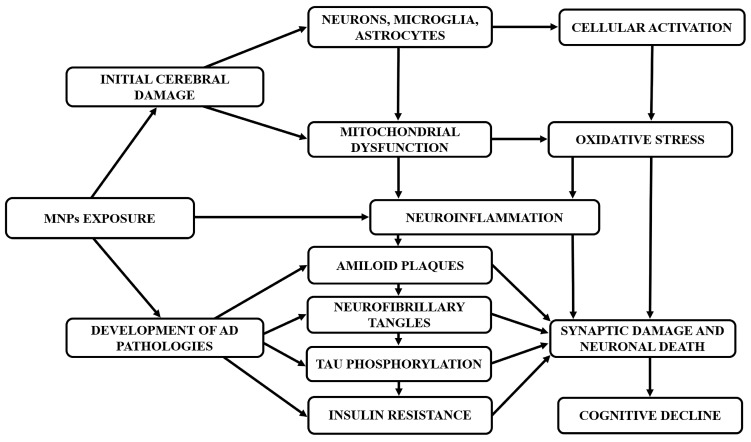
Schematic diagram of the molecular mechanism hypothesizing that impaired insulin signaling in the brain is responsible for early and progressive cognitive defects.

Oxidative stress, involving the production of reactive oxygen and nitrogen species, is constantly produced in cells. Normal physiological processes are maintained by a balance of cellular ROS/RNS, whereas an excess of ROS/RNS is dangerous to intracellular molecules and is correlated with chronic diseases such as cardiovascular disease, diabetes, inflammatory diseases, and neurological disorders, including AD and PD [194]. In AD, oxidative stress is a trigger and a factor that accelerates its progression (Figure 6) [195]. The disease is characterized by the abnormal deposition of Aβ peptides and the accumulation of neurofibrillary tangles composed of hyperphosphorylated tau protein, culminating in dementia. Studies show that oxidative stress increases the aggregation and production of Aβ peptide, as well as stimulates phosphorylation of tau protein, both of which lead to neurotoxicity (Figure 6). In turn, the accumulation of Aβ peptide and tau protein contributes to a redox imbalance, stimulating mitochondrial dysfunction and increasing ROS production.

Loss of Nrf2 in transgenic AD mouse models has shown increased levels of Aβ peptide and phosphorylated tau protein, resulting in neurotoxicity [196]. In PD, the degeneration of dopamine neurons in the cerebral substantia nigra is a distinctive feature, and oxidative stress is considered a cause of dopaminergic neurotoxicity. Dopaminergic neuronal loss in PD is significantly related to ROS production, which results from low GSH levels, high iron and calcium levels, and dopamine metabolism [197,198]. Skibinski et al. showed that Nrf2 reduces toxicity induced by LRRK2 (α-synuclein and leucine-rich repeat kinase 2), maintaining neuronal protein homeostasis. Nrf2 is also involved in the clearance of synuclein and the conversion of LRRK2 aggregation into inclusion bodies, leading to a reduction in neuronal toxicity. Furthermore, when the immune system is activated at a later time, the NF-κB protein releases inflammatory substances such as IL-6, TNF-α, and IL-1β. These substances can damage nerve cells, causing neurotoxicity. To neutralize this damage, melatonin, a natural antioxidant in our body, offers protection to neurons. It does this by triggering the Nrf2 pathway and reducing nerve cell death, as demonstrated in HT22 cells [199]. The Nrf2/ARE network and its intricate relationship with epigenetic mechanisms represent a dynamic field with great therapeutic potential for neurodegenerative disorders by modulating stress resilience responses. Further investigations, including human studies, will be critical to translate promising findings from research into effective clinical approaches.

## 7. Applications of Next Generation Sequencing in Chronic Diseases

Next-generation sequencing (NGS) has revolutionized biomedical research by enabling high-throughput, comprehensive exploration of genomic, transcriptomic, and epigenetic landscapes across a wide range of physiological and pathological conditions [200,201,202,203]. While their initial applications were largely focused on oncology, NGS technologies have increasingly been adopted to investigate the molecular mechanisms of chronic diseases, such as neurodegenerative diseases [204], metabolic syndromes [205], and autoimmune conditions [206]. In particular, transcriptomic profiling through RNA sequencing (RNA-seq) has provided crucial insights into dysregulated pathways and cell-type-specific responses in diseases like AD [207,208], autism spectrum disorders (ASD) [209], and diabetes mellitus [210], revealing novel biomarkers and therapeutic targets [211]. Regarding environmental pollutants, a recent study using RNA-seq has shown that long-term exposure to PS-NMPs of 0.5 µm and 5 µm diameter can induce liver inflammation and fibrosis by targeting *Acot3*, *Abcc3,* and *Nr1i3* genes in experimental models [212]. Furthermore, an interesting study performed by Dragacevic et al. using NGS identified novel probiotic bacteria strains such as *H. alvei UUNT_MP41* and *H. paralvei UUNT_MP29* isolated from the gut microbiota of common carp that showed significant resistance to various antibiotics and the peculiar ability to biodegrade MPs [213]. The analysis also demonstrated that *H. alvei UUNT_MP41* and *H. paralvei UUNT_MP29* possess the gene encoding the ATP-dependent chaperone *ClpB* (heat shock protein) that confers probiotic properties associated with MP degradation [213]. Beyond clinical pathologies, NGS has also emerged as a powerful tool to study the biological effects of nutritional supplements and plant-derived polyphenols [214]. These molecules engage in complex interactions with host gene expression [215], microbiota composition [216], and signaling pathways [217], which can be analyzed at a systems level using omics-based approaches. Multi-omics strategies integrating NGS data have enabled a deeper understanding of how dietary components influence cellular homeostasis [218], immune modulation [219], and oxidative stress responses [220], paving the way for precision nutrition and functional food research. This section highlights recent advances in the application of NGS to both disease modeling and the mechanistic study of functional compounds, emphasizing its versatility as a bridge between molecular biology, personalized medicine, and nutritional science (Table 7).

### 7.1. Next-Generation Sequencing and the Molecular Dissection of the NRF2 Pathway

The implementation of NGS has been instrumental in elucidating redox-sensitive signaling networks, particularly those involving the *NFE2L2* gene and its key regulators KEAP1 and CUL3. These molecules govern cellular responses to oxidative stress and ferroptosis, and their dysregulation is implicated in fibrosis, cancer, and neurodegeneration. For instance, Zhu et al. applied single-cell RNA sequencing (scRNA-seq) to fibroblasts from arthrofibrosis patients, uncovering ferroptosis-related transcriptional signatures and suggesting a cytoprotective role for Nrf2 against ROS-induced ferroptosis [221]. Similarly, in multifocal hepatocellular carcinoma (HCC), Amemiya et al. used targeted NGS on an Ion Torrent platform to reveal heterogeneity in KEAP1/NRF2 mutations between tumor lesions, pointing to molecular evolution as a driver of therapy resistance [222]. In another translational application, Guan et al. employed amplicon-based NGS to show that *NFE2L2/KEAP1/CUL3* mutations correlate with poorer disease-free survival and higher recurrence in glottic squamous cell carcinoma treated with radiotherapy, highlighting their potential as predictive biomarkers [223]. NGS combined with bioinformatics has also illuminated the role of ferroptosis in other conditions. In PD, Jian et al. integrated multiple transcriptomic datasets and identified a panel of ferroptosis-related genes, some of which (e.g., DDIT4, RELA, CAV1) show prognostic potential [224]. In ischemic cardiomyopathy, Liu et al. pinpointed ferroptosis-associated differentially expressed genes (i.e., *TFRC*, *SCD*, *SLC2A1*, *EGR1*, *GDF15*, *SNCA*, *PLIN2*, and *NQO1*), suggesting novel cardiac biomarkers [225]. In HCC, Feng et al. identified SEH1L as a regulator of tumor growth through ferroptotic pathways involving ATF3/HMOX1/GPX4, confirmed by in vitro and in vivo experiments [226]. Finally, a recent study performed by Liao et al. using NGS analyzed differentially expressed genes, including *Nrf2*, *Hmox-1*, and *GPX4* genes, in rats with acute compartment syndrome. Specifically, treatment with minocycline at a concentration of 40 and 80 mg/kg effectively inhibits ferroptosis by activating the Nrf2/Hmox-1/GPX4 pathway [227]. Collectively, these studies underscore the power of NGS—particularly scRNA-seq and targeted panels—in mapping the transcriptional and mutational landscapes of the NRF2-ferroptosis axis across multiple disease settings, offering insights into pathogenesis, prognosis, and potential therapeutic targets.

### 7.2. NGS in Neurological Diseases

NGS has broadened our molecular understanding of complex chronic diseases. In AD, Mitsumori et al. utilized methylation capture sequencing on peripheral blood to identify differentially methylated regions (DMRs) in genes such as ANKH and MARS [228]. When combined with APOE genotyping, these markers improved diagnostic accuracy, supporting minimally invasive biomarker development. Pagano et al. compared short-read and long-read sequencing platforms in AD epigenomics, highlighting trade-offs in resolution and cost. They emphasized the potential of long-read approaches (e.g., Oxford Nanopore) to resolve complex methylation patterns relevant to AD and other dementias [229]. NGS is also pivotal in diagnosing ASD and neurodevelopmental disorders (NDDs). In a cohort of 868 children, Neuens et al. achieved a 27% diagnostic yield through clinical exome sequencing, with higher rates in syndromic or developmentally delayed cases. Stratification improved further with copy number variant (CNV) analysis [230]. Ji et al. explored alterations in the 17p13.3 region, identifying disease-associated variants in PAFAH1B1, YWHAE, and CRK, suggesting the value of targeted genomic screening for precise subtyping of NDDs [231]. These findings highlight the versatility of NGS—from bulk and single-cell transcriptomics to epigenomics and mtDNA sequencing—in uncovering novel disease mechanisms, stratification markers, and targets for early intervention across diverse chronic pathologies.

### 7.3. Polyphenols and Their Bioactivity Explored Through Multi-Omics Approaches

Polyphenols, known for their antioxidant and anti-inflammatory properties, have been increasingly studied through NGS and multi-omics approaches to better understand their molecular impact. Huang et al. applied NGS to profile small RNAs in Matcha green tea, revealing a rich spectrum of plant-derived miRNAs (e.g., lja-miR166-3p, csn-miR396d-5p) correlated with catechin and amino acid content. These miRNA profiles varied across cultivars and seasons, and high-temperature extraction (95 °C) enhanced miRNA yield, suggesting dietary polyphenols may exert effects through plant miRNAs [232]. Yu et al. adopted a combined transcriptomic and metabolomic approach to characterize “purple coffee,” a Coffea arabica variety with high anthocyanin levels. They identified the upregulation of phenylpropanoid biosynthesis genes and increased flavonoid content. Additionally, 16S rRNA sequencing of the phyllosphere microbiome revealed distinct bacterial taxa (e.g., Methylobacterium, Comamonas), suggesting polyphenols may modulate microbial communities and contribute to stress resilience [233]. At the cellular level, Kimsa-Dudek et al. used RNA sequencing to assess the transcriptomic response of human dermal fibroblasts to caffeic acid (CA). Over 1000 genes were differentially expressed, particularly those involved in apoptosis and stress response. Co-treatment with a static magnetic field (SMF) further modulated gene expression, including marked upregulation of heat shock proteins HSPA6 and HSPA7, indicating a synergistic effect of CA and physical stimuli in activating cytoprotective pathways [234]. Together, these findings highlight the power of NGS and integrative omics in unraveling the multifaceted roles of polyphenols—from their bioavailability and regulatory roles in gene expression to their influence on host–microbiota interactions—emphasizing their potential in personalized nutrition and therapeutic strategies.

**Table 7:** Summary of recent applications of NGS technologies in disease models and nutritional studies. Techniques include transcriptomic, epigenomic, and mitochondrial sequencing, highlighting their utility in biomarker discovery, mechanistic insight, and patient stratification.

Technique	Disease/Application	Application	Ref.
scRNA-seq	Arthrofibrosis	Identification of ferroptosis-related signatures and the cytoprotective NRF2 role	[221]
Targeted NGS (Ion Torrent)	Multifocal HCC	Analysis of KEAP1/NRF2 mutation heterogeneity	[222]
Amplicon-based NGS	Glottic carcinoma	*NFE2L2*/KEAP1/CUL3 mutations as prognostic markers	[223]
Meta-analysis of RNA-seq	Parkinson’s disease	Identification of ferroptosis-related prognostic genes	[225]
RNA-seq + functional assays	HCC	SEH1L as ferroptotic regulator via ATF3/HMOX1/GPX4 axis	[226]
Methylation capture sequencing	Alzheimer’s disease	DMRs in ANKH and MARS genes; improved diagnostics with APOE genotyping	[228]
Long-read sequencing	Alzheimer’s disease	Mapping complex methylation patterns	[229]
Clinical exome + CNV analysis	Autism/NDD	27% diagnostic yield, enhanced with CNVs	[230]
Targeted sequencing of 17p13.3	Neurodevelopmental disorders	Variants in PAFAH1B1, YWHAE, and CRK genes	[231]
Mitochondrial DNA sequencing	Type 2 diabetes	SNPs in MT-ND5 and MT-ATP6 linked to insulin resistance	[235]
RNA-seq	Diabetic nephropathy	Differentially expressed genes in mitochondrial and inflammatory pathways	[236]
scRNA-seq	Diabetic kidney	Tubule-specific pro-inflammatory gene signatures	[237]
Small RNA-seq	Matcha (nutritional study)	Detection of plant-derived miRNAs influenced by cultivar and temperature	[232]
Transcriptomics + Metabolomics	Anthocyanin-rich coffee	Upregulated flavonoid biosynthesis and microbiome modulation	[233]
RNA-seq	Human fibroblasts + caffeic acid	HSP expression modulated by static magnetic field + CA	[234]
mRNA/miRNA-seq	Brain endothelial cells (BBB)	Creation of the BBBomics database for systems biology research	[238]
RNA-seq with standardized workflow	BBB and CSF barriers	BtRAIN guidelines for barrier transcriptomics	[239]
snRNA-seq	Schizophrenia	Gene expression alterations in pericytes and ependymal cells	[240]
Whole-exome sequencing	Coats disease	Rare variants in BRB-related genes (HMCN1, NPHP4)	[241]
NGS	Acute compartment syndrome	Identify differentially expressed genes (Nrf2, Hmox-1, and GPX4) to inhibit ferroptosis in vivo	[227]

## 8. Conclusions

The interaction between MNPs exposure and the development of metabolic and brain disorders is an evolving field of research, placing particular emphasis on the role of functional nutrients in mitigating these effects on human health. After absorption through the gastrointestinal tract, MNPs can unknowingly accumulate in various tissues, such as the liver, spleen, immune system, and nervous system, causing cytotoxicity, inflammation, and genetic damage, potentially contributing to the onset and progression of AD. Certain functional nutrients, such as polyphenols, flavonoids, phenylpropanoids, phenolic acids, diterpenoids, and triterpenoids, represent a promising therapeutic strategy targeting the *NFE2L2* gene and resilience proteins as a cellular defense mechanism to inhibit MNP-induced oxidative stress, neuroinflammation, and ferroptosis, leading to aberrant insulin signaling, tau hyperphosphorylation, and Aβ accumulation, ultimately causing neuronal death. The review examined several functional nutrients, including ursolic acid, verbascoside, tanshinones, cynarin, and baicalein, which have demonstrated neuroprotective potential and a remarkable ability to counteract some aspects of AD pathogenesis often referred to as “type 3 diabetes” or brain insulin resistance. Finally, by activating the Nrf2 pathway, these nutrients modulated the regulation of inflammatory pathways, particularly mitochondrial function and neurogenesis. Furthermore, the *NFE2L2* gene and related pathway are epigenetically modulated, influencing gene expression through alterations, such as DNA methylation and histone modification, which have profound implications for cellular resilience response and disease prevention. In conclusion, this review highlights the importance of a personalized nutritional approach to counteract the harmful effects of MNPs and improve clinical outcomes in AD patients. Identifying functional nutrients that activate the Nrf2 pathway represents a promising avenue for developing preventive and therapeutic interventions. Innovation in this field, supported by advances in in vitro platforms, opens new perspectives for more precise and patient-centered medicine.

## Data Availability

No new data were created or analyzed in this study. Data sharing is not applicable to this article.
